# Differential regulation of polarized synaptic vesicle trafficking and synapse stability in neural circuit rewiring in *Caenorhabditis elegans*

**DOI:** 10.1371/journal.pgen.1006844

**Published:** 2017-06-21

**Authors:** Naina Kurup, Dong Yan, Karina Kono, Yishi Jin

**Affiliations:** 1 Neurobiology Section, Division of Biological Sciences, University of California, San Diego, La Jolla, California, United States of America; 2 Howard Hughes Medical Institute, University of California, San Diego, La Jolla, California, United States of America; 3 Department of Cellular and Molecular Medicine, University of California, San Diego, La Jolla, California, United States of America; University of California San Francisco, UNITED STATES

## Abstract

Neural circuits are dynamic, with activity-dependent changes in synapse density and connectivity peaking during different phases of animal development. In *C*. *elegans*, young larvae form mature motor circuits through a dramatic switch in GABAergic neuron connectivity, by concomitant elimination of existing synapses and formation of new synapses that are maintained throughout adulthood. We have previously shown that an increase in microtubule dynamics during motor circuit rewiring facilitates new synapse formation. Here, we further investigate cellular control of circuit rewiring through the analysis of mutants obtained in a forward genetic screen. Using live imaging, we characterize novel mutations that alter cargo binding in the dynein motor complex and enhance anterograde synaptic vesicle movement during remodeling, providing *in vivo* evidence for the tug-of-war between kinesin and dynein in fast axonal transport. We also find that a casein kinase homolog, TTBK-3, inhibits stabilization of nascent synapses in their new locations, a previously unexplored facet of structural plasticity of synapses. Our study delineates temporally distinct signaling pathways that are required for effective neural circuit refinement.

## Author summary

In this study, we identify pathways that regulate the formation and maintenance of synapses, the functional connections between neurons, in the nervous system of the nematode *C*. *elegans*. Our work characterizes the interaction between molecular motors kinesin and dynein, which carry cargo and move towards opposite ends of microtubules during synapse formation. We also address the role of a protein kinase gene TTBK-3 in maintaining synapse structure once synaptic components have reached the sites of new synapses. Our findings shed mechanistic insight into the coordination of molecular motors and the cytoskeleton in neural circuit function.

## Introduction

Neurons communicate through synapses, necessitating a system of checks and balances to achieve precise patterns of synaptic connectivity that execute neural circuit function. Large scale axonal growth and pruning mediate synapse formation with appropriate targets during development, shaping neuronal circuits during critical periods of plasticity [1]. Hyper- and hypo- connectivity in different brain regions is a widely observed phenomenon in children with autism spectrum disorders (ASDs) and related comorbid conditions [2]. Brain development defects during critical postnatal periods of plasticity are also thought to contribute to schizophrenia, which has a varying age of onset [3]. Structural synaptic plasticity is not purely a developmental phenomenon-synapse remodeling occurs in both normal and diseased adult brains in various contexts [4, 5]. The mechanisms underlying synapse assembly and elimination have thus been the subject of intense study for several decades, although a majority of experimental models focused on synaptic plasticity that is coupled to neurite outgrowth and retraction [6, 7]. With recent advances in *in vivo* imaging techniques, instances of synaptic rewiring that are independent of large scale neurite rearrangement have been identified in the mammalian central nervous system [4, 8]. Elucidating the mechanisms underlying the cellular dynamics of such refinement, particularly in pre-synaptic terminals, is of general significance.

**Fig 1:**
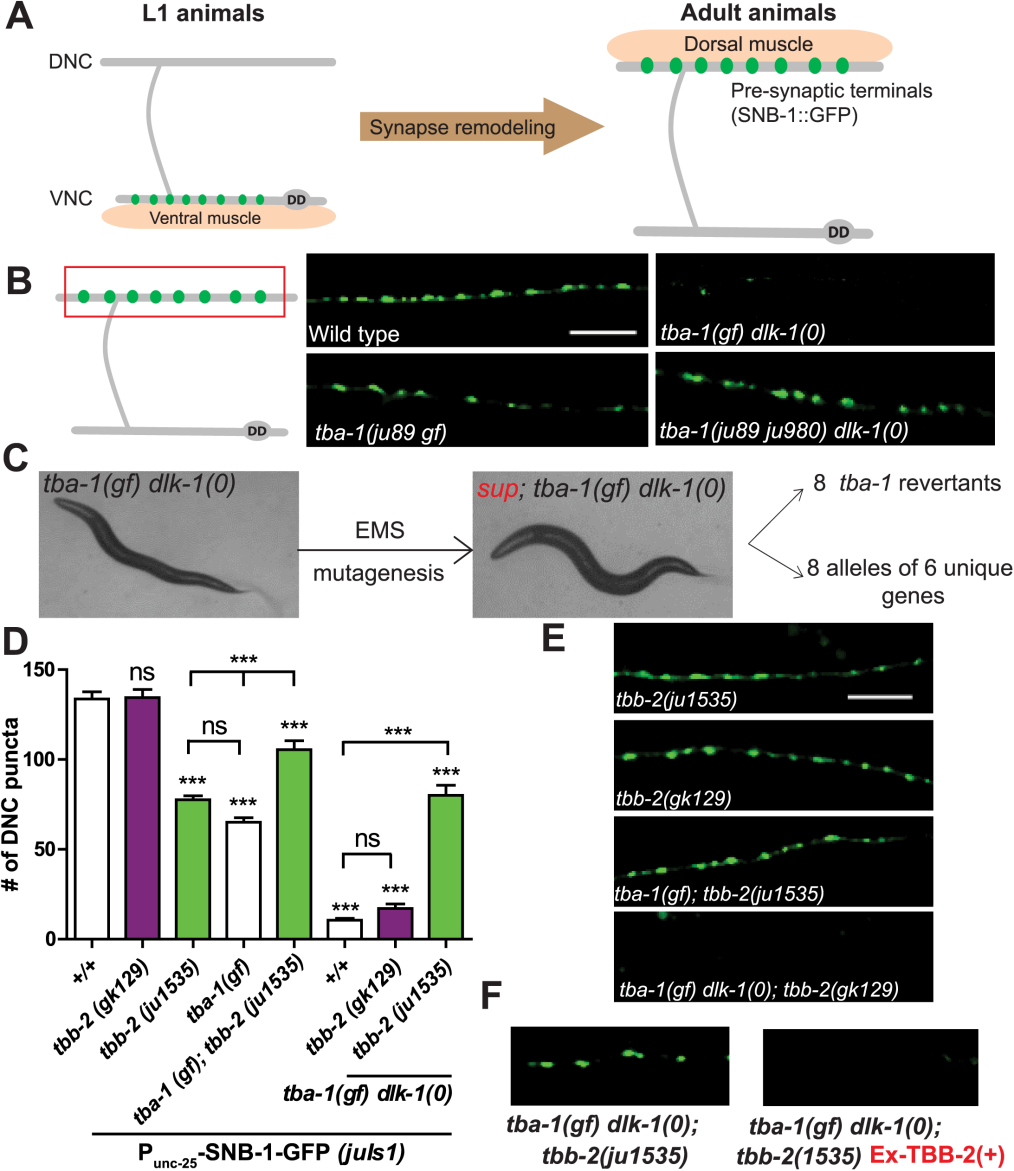
Intragenic mutations in *tba-1* and a novel *tbb-2* mutation suppress synapse remodeling defects in *tba-1(gf) dlk-1(0)*. (A) Schematic of remodeling of DD neuron synapses. In young larvae, pre-synaptic terminals are visualized along the ventral nerve cord (VNC) using GFP- tagged synaptobrevin (SNB-1::GFP). These synapses are completely eliminated in wild type animals, to form new synapses along the dorsal nerve cord (DNC).(B) Schematic of an adult DD neuron, with the red box representing the region of interest. Representative images of DD synapses in the adult DNC of various genotypes, visualized using *P*_*unc-25*_*-*SNB-1::GFP (*juIs1*). Scale bar: 10 μm. (C) Bright field images of a *tba-1(gf) dlk-1(0)* animal and an animal isolated following EMS mutagenesis of *tba-1(gf) dlk-1(0)*. Suppressors were isolated based on improved behavior, with a total of 8 intragenic *tba-1* mutants and 8 suppressors with mutations in genes besides *tba-1* and *dlk-1*. (D) Representative images of DD synapses in the adult DNC of various genotypes, visualized using *P*_*unc-25*_-SNB-1::GFP (*juIs1*). Scale bar: 10 μm. (E) Quantification of synaptic puncta in the DNC of adult animals. Data are mean ± SEM; n>10 animals per genotype. Statistics: One-Way ANOVA followed by Tukey’s posttest; ***p<0.001, ns- not significant. (F) DNC synapses in *tba-1(gf) dlk-1(0); tbb-2(ju1535); juIs1* animals that either lack or contain a rescuing transgene expressing wild type TBB-2 (Ex-TBB-2(+)).

In the *C*. *elegans* locomotor circuit, a subset of type-D GABAergic motor neurons exhibit critical period synapse plasticity. Upon birth and in young larvae, the Dorsal D (DD) neurons initially form synapses with ventral body wall muscles. During an early developmental molt, these early synapses are disassembled, and new synapses are formed with dorsal body wall muscles, without overt changes in neuronal morphology (Fig 1A) [9]. DD synapse remodeling is developmentally stereotyped, activity dependent, and uncoupled from neurite outgrowth, providing a tractable genetic framework to study the molecular mechanisms underlying structural synaptic plasticity. Numerous studies have provided insights into the conserved transcriptional programs that regulate the initiation of DD synapse remodeling and that maintain the temporal precision of synapse remodeling (reviewed in [10]).

Once circuit connectivity changes have been initiated in the DD neurons, the cellular execution of synapse assembly and disassembly takes place. Pre-synaptic terminals are eliminated from the DD ventral neurite, following which synaptic vesicles are transported to the DD dorsal neurite where they assemble to form new synapses that are stable for the lifetime of the animal. Previous work from our lab and others has found that dynamic microtubules (MTs) are required for synaptic vesicle transport to DD dorsal neurite during remodeling [11] and the patterning of new pre-synaptic terminals is achieved by the sequential action of anterograde and retrograde motors Kinesin-3/UNC-104 and dynein, respectively [12]. Synapse elimination from the DD ventral neurite is mediated in part by the cyclin Y homolog CYY-1 and the apoptotic cell death pathway [12, 13].

In this study, we characterized multiple mutants isolated from a genetic screen for genes involved in DD synapse remodeling. We performed this screen on a mutant strain containing a gain-of-function (*gf)* mutation of alpha-tubulin *tba-1*, and a loss-of-function (*0)* mutation of the conserved MAPKKK *dlk-1*. This *tba-1(gf) dlk-1(0)* double mutant combination results in defective DD synapse remodeling due to a reduction in MT dynamics [11]. We identified mutations in the *C*. *elegans* α- and β-tubulin genes *tba-1* and *tbb-2* that reversed defects in MT architecture. We also show that novel mutations in the minus end directed motor dynein and its adaptor protein dynactin ameliorate defects in kinesin-mediated synaptic vesicle transport to the DD dorsal neurite during remodeling, highlighting the interdependence of the two motors even in cases of polarized cargo movement. We further find that a member of the casein kinase superfamily, TTBK-3, specifically acts after remodeling is complete to modulate nascent synapse stability on the dorsal neurite, a previously uncharacterized aspect of synaptic plasticity. Our observations indicate that the dynein motor complex and TTBK-3 act at distinct temporal windows to differentially regulate synapse rewiring.

## Results

### A forward genetic screen to identify novel regulators of DD synapse remodeling

A missense mutation in *C*. *elegans* α-tubulin, *tba-1(ju89)* (henceforth *tba-1(gf)*) results in a mild reduction in the synapse number of GABAergic motor neurons and a dampening of the amplitude of sinusoidal locomotion (Fig 1B) [11, 14]. In animals carrying both *tba-1(gf)* and a loss of function of the conserved MAPKKK DLK-1 (*dlk-1(0)*), DD remodeling is completely blocked such that the dorsal neurites contain almost no synapses, when visualized by the synaptic marker *juIs1* (*P*_*unc-25*_-SNB-1-GFP) (Fig 1B) [11]. Consistent with a lack of GABAergic innervation, *tba-1(gf) dlk-1(0)* double mutant animals are uncoordinated and coil dorsally when touched in the head (Fig 1C) [11]. We performed a suppressor screen on *tba-1(gf) dlk-1(0); juIs1* animals, first based on behavioral improvement, then by visual examination of synapses in DD neurons. We mapped the suppressor mutations to various genetic loci using whole genome sequencing and subsequent recombinant mapping (see methods). Below, we report the characterization of these mutations.

### Intragenic mutations of *tba-1* and a novel mutation of *tbb-2* ameliorate the activity of *tba-1(gf)*

*tba-1(gf)* alters a conserved glycine residue to arginine (G414R) in the C-terminal H11-12 loop of α-tubulin (S1 Fig) [14]. This C-terminal domain of α-tubulin is implicated in microtubule associated protein (MAP) binding [15], and the G414R mutation resulted in MTs that are misoriented at both synaptic and asynaptic sites along the axonal processes of DD neurons [11]. *tba-1(0)* null mutants appeared wild-type, presumably due to functional redundancy among the nine α-tubulin homologs in *C*.*elegans* [11, 14]. Double mutant animals of *tba-1(0)* and *dlk-1(0)* are also superficially normal, indicating that *tba-1(gf)* acts synergistically with *dlk-1(0)* to produce defective synapse remodeling [11]. We found eight mutants that fully suppressed the uncoordinated behavior of *tba-1(gf) dlk-1(0)* and were completely normal with regards to synapse formation and DD synapse remodeling. DNA sequence analyses *of tba-1* revealed that these suppressors contained additional mutations besides the *ju89* nucleotide change, and therefore were classified as intragenic revertants of *tba-1(gf)* (Fig 1C). Three suppressors (*ju964*, *ju966* and *ju975)* caused nonsense mutations at different amino acids of *tba-1* (S1 Fig and S1 Table). This suggests that truncated versions of TBA-1 produced in these mutants were likely not functional. One missense mutation at the start codon ATG (*ju980*) also behaved similar to *tba-1(0)*, with synapse formation and locomotion restored to normal in *tba-1(ju980 ju89) dlk-1(0)* animals (Fig 1B, S1 Fig). The other four suppressors caused missense mutations at highly conserved regions of α-tubulin (S1 Fig and S1 Table). Two missense mutations (*ju973* (L426F) and *ju987* (A419T)) were in H12 of the C-terminal domain, and could possibly have reversed the MAP binding defects caused by *tba-1(gf)* (S1 Fig). Another missense mutation, *ju965* (S138L), was close to the GTP binding pocket of α-tubulin [15] (S1 Fig). The last missense mutation, *ju962* (S285F), was in the intermediate domain of α-tubulin, reported to be necessary for binding of the MT stabilizing drug, taxol (Fig 1D and S1 Fig) [15]. Mutations in the intermediate domain or the GTP binding pocket possibly prevented the incorporation of *tba-1* into MT polymers. While we cannot exclude the possibility that these mutations might also reduce TBA-1 protein levels, our results highlight the *in vivo* importance of specific residues within functional domains of α-tubulin.

The *C*. *elegans* genome encodes six β-tubulin genes that function partially redundantly in various tissues of the organism and at different developmental stages [16–19]. We mapped the suppressor *ju1535* to β-tubulin *tbb-2*, causing a conserved Proline305 to Serine change (P305S) in the intermediate domain, which lies in the internal surface of the MT polymer (S1 Fig). *tbb-2 (ju1535)* partially suppressed the behavioral and synapse remodeling defects of *tba-1(gf) dlk-1(0)* (S1 Table and Fig 1D), and such suppression was rescued by transgenic expression of wild type TBB-2 in *tba-1(gf) dlk-1(0); tbb-2(ju1535)* animals (Fig 1F). Interestingly, *tbb-2 (ju1535)* single mutant animals were smaller than wild type animals, and displayed a significant reduction in DD neuron synapse number, similar to *tba-1(gf)* animals (Fig 1D and 1E). Since *tba-1* and *tbb-2* form heterodimers in the *C*. *elegans* embryo, and have overlapping neuronal expression patterns [18–20], we hypothesized that *tbb-2(ju1535)* might suppress *tba-1(gf)* alone. Indeed, *tba-1(gf); tbb-2(ju1535)* double mutant animals displayed increased DD neuron synapse numbers compared to either single mutant, albeit fewer than those seen in wild type animals (Fig 1D and 1E).

*tbb-2(ju1535)* behaved as a neomorphic allele, as *gk129*, a null *(0)* mutation of *tbb-2* did not cause overt defects in locomotion or synapse formation and also did not suppress the synapse remodeling defects of *tba-1(gf) dlk-1(0)* animals (S1 Fig, Fig 1D and 1E). *tba-1(gf); tbb-2(0)* animals were viable and displayed similar synapse formation defects to *tba-1(gf)* single mutant animals, whereas *tba-1(0); tbb-2(0)* animals were lethal due to their requirement in early embryonic development [14]. Taken together, these results suggest that while heterodimers of TBA-1 and TBB-2 were essential for embryonic development, in the absence of *tbb-2*, *tba-1(gf)* could form heterodimers with other β-tubulins that were then incorporated into MTs. *tba-1(gf)* resulted in a change in MT architecture [11] and is a mutation altering the external surface of MTs, separate from domains responsible for GTP binding and heterodimer formation (S1 Fig) [14, 15]. Thus, the suppression of *tba-1(gf)* by *tbb-1(ju2535)* may likely be through either modifying or reducing the incorporation of *tba-1(gf); tbb-2(ju1535)* heterodimers in MTs, in turn reducing the number of abnormal MTs.

### Dynein-dynactin complex function is important for synapse remodeling

**Fig 2:**
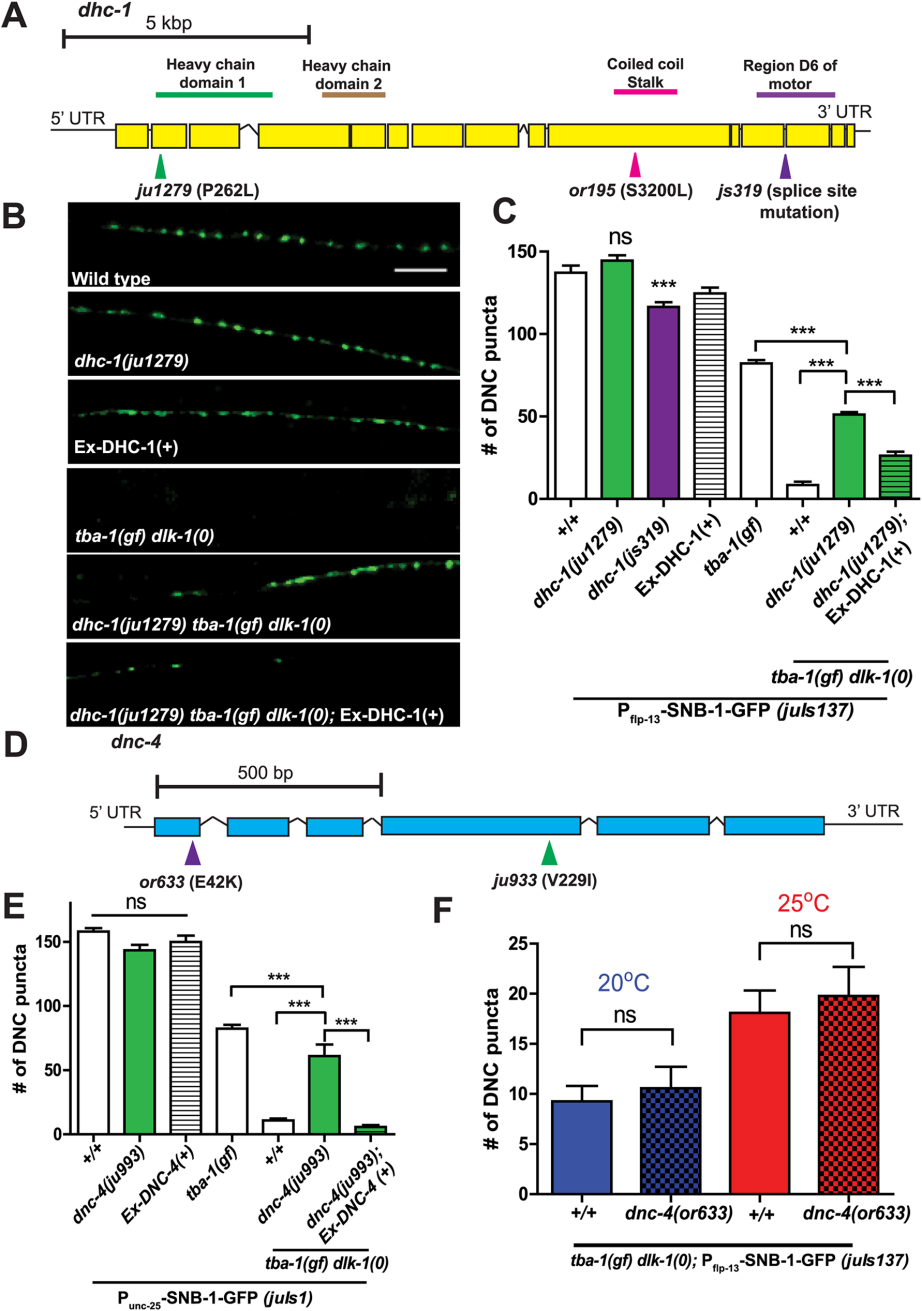
*ju1279* and *ju993* are novel alleles of *dhc-1* and *dnc-4*, respectively. (A) Gene structure of *dhc-1*, with *ju1279* and reference alleles *or195* and *js319* marked. *ju1279* alters the N-terminal Heavy chain domain 1, *or195* alters the coiled coil stalk and *js319* alters region D6 of the motor. (B) Representative images of DD synapses along the DNC (*P*_*flp-13*_-SNB-1::GFP (*juIs137*)) in adult animals. Ex-DHC-1 denotes extrachromosomal copies of wild type DHC-1. Scale bar: 10 μm. (C) Quantification of synaptic puncta in the DNC of adult animals. Data are mean ± SEM; n>10 animals per genotype. Statistics: One-Way ANOVA followed by Tukey’s posttest; ***p<0.001, ns- not significant. (D) Gene structure of *dnc-4*, with *ju933* and the reference allele *or633* also marked. (E) Quantification of synaptic puncta in the DNC (*P*_*unc-25*_-SNB-1::GFP (*juIs1*)) of adult animals. Ex-DNC-4(+) denotes extrachromosomal copies of wild type DNC-4. Data are mean ± SEM; n>8 animals per genotype. Statistics: One-Way ANOVA followed by Tukey’s posttest; ***p<0.001, ns- not significant. (F) Quantification of synaptic puncta in the DNC (*P*_*flp-13*_-SNB-1::GFP (*juIs137*)) of adult animals. Animals were cultured at two different temperatures, the permissive (20^°^C) and restrictive (25^°^C) temperatures for *or633*, starting from late L1. Data are mean ± SEM; n>8 animals per genotype. Statistics: One-Way ANOVA followed by Tukey’s posttest; ns- not significant.

We mapped one of the suppressors, *ju1279*, to the *C*. *elegans* cytoplasmic dynein heavy chain, *dhc-1*. Cytoplasmic dynein is a large multi-subunit molecular motor that comprises of two catalytic heavy chains, as well as numerous light and intermediate chains. Dynein moves towards MT minus ends and is the primary motor involved in retrograde axonal transport, with mutations in dynein and its adaptor proteins implicated in several neurodegenerative disorders [21]. *ju1279* converts a conserved proline residue to leucine (P262L) in the N-terminal region 1 of the tail domain of DHC-1 (Fig 2A and S2 Fig), which is responsible for dynein homodimerization and acts as a scaffold for subunit assembly [22]. *dhc-1(ju1279)* acted as a weak suppressor of *tba-1(gf) dlk-1(0)*, since synapse remodeling was only partially restored in *dhc-1(ju1279) tba-1(gf) dlk-1(0)* triple mutant animals, with a significant reduction in dorsal neurite synapse number compared to *tba-1(gf)* animals (Fig 2B and 2C). We confirmed that *dhc-1(ju1279)* was causative by rescuing the suppression of *tba-1(gf) dlk-1(0)* using extra-chromosomal copies of a fosmid containing the full genomic region of DHC-1 (Fig 2B and 2C).

To understand how the *ju1279* allele affects *dhc-1* function, we examined two well characterized alleles of *dhc-1*, *or195* [23] and *js319* [24]. *or195* is a conserved serine to leucine change in the MT binding stalk region of DHC-1, resulting in temperature sensitive lethality (Fig 2A), while *js319* is a splice site mutation in the C-terminal conserved motor domain of DHC-1 [25], producing viable but visibly dumpy animals at 25^°^C (Fig 2A, S2 Fig). In contrast, *dhc-1(ju1279)* animals appeared superficially wild type at 25^°^C, as did *dhc-1(ju1279)/dhc-1(js319)* and *dhc-1(ju1279)/dhc-1(or195)* heterozygous animals (S2 Fig). *dhc-1(js319)* animals also had reduced DD synapse numbers (Fig 2D) while overexpression of wild type DHC-1 or *dhc-1(ju1279)* did not alter synapse number in adult animals (Fig 2C and 2D). Additionally, triple mutants of *dhc-1(js319)* or *dhc-1(or195)* with *tba-1(gf) dlk-1(0)* were embryonic lethal even at temperatures lower than 25^°^C. Taken together, these results suggest that *ju1279* is a novel allele of *dhc-1*, uniquely altering dynein function in the context of synapse remodeling.

Another suppressor, *ju993*, changed valine 229 to isoleucine in the *C*. *elegans* p62 subunit of dynactin DNC-4 (Fig 2D). Dynactin is a large multi-subunit protein complex that is essential for most cellular functions of cytoplasmic dynein, including MT binding and linking dynein to its cargo during fast axonal transport [26–29]. DNC-4, together with p25 and p27 subunits, interacts with actin-related proteins Arp1 and Arp11 to form the pointed end of the dynactin complex, which is positioned diametrically opposite dynein motor domains to primarily influence cargo binding [28–30]. In *dnc-4(ju993)* adult animals DD neurons formed synapses in the dorsal neurites, and the pattern and number of synapses were comparable to wild type. In *tba-1(gf) dlk-1(0)* animals, *dnc-4(ju993)* significantly increased the number of synapses in DD dorsal neurites (Fig 2E). Expression of wild type copies of DNC-4(+) rescued the suppression of *tba-1(gf) dlk-1(0)* by *dnc-4(ju933)* (Fig 2E). *dnc-4(ju933)* complemented the temperature-sensitive embryonic lethality of *dnc-4(or633)*, which altered a conserved glutamic acid to lysine in the N-terminal region (S2 Fig) [31]. Additionally, *dnc-4(or633)* did not suppress the synapse remodeling defects of *tba-1(gf) dlk-1(0)* at the permissive temperature (20^°^C), or when shifted to the restrictive temperature (25^°^C) after embryonic development (Fig 2F), indicating that *ju993* is a novel mutation of *dnc-4*. In conjunction with the effects on SV transport seen in *dhc-1(ju1279)* animals, and the similar levels of suppression seen in both *dhc-1* and *dnc-4* alleles (Fig 2C and 2E), we propose that altering dynein-dynactin complex interactions has a profound effect synapse remodeling.

### Altered dynein activity promotes anterograde vesicle transport during remodeling

We next sought to understand the mechanism by which *dhc-1(ju1279)* suppressed the synapse remodeling defects of *tba-1(gf) dlk-1(0)*. The remodeling defects in *tba-1(gf) dlk-1(0)* animals were primarily brought about by an increase in MT stability, resulting in reduced synaptic vesicle (SV) transport in the DD neurons during remodeling [11]. We first considered the possibility that *dhc-1(ju1279)* modifies MT dynamics in *tba-1(gf) dlk-1(0)*, since an established role of cytoplasmic dynein is to stabilize dynamic MT plus ends by tethering them to the cell cortex [32]. However, *dhc-1(ju1279)* had no significant effect on the number of dynamic MTs or their direction of growth in both wild type and *tba-1(gf) dlk-1(0)* adults (S2 Fig), leading us to conclude that the suppression of synapse remodeling defects by *dhc-1(ju1279)* did not result from a change in MT dynamics.

**Fig 3:**
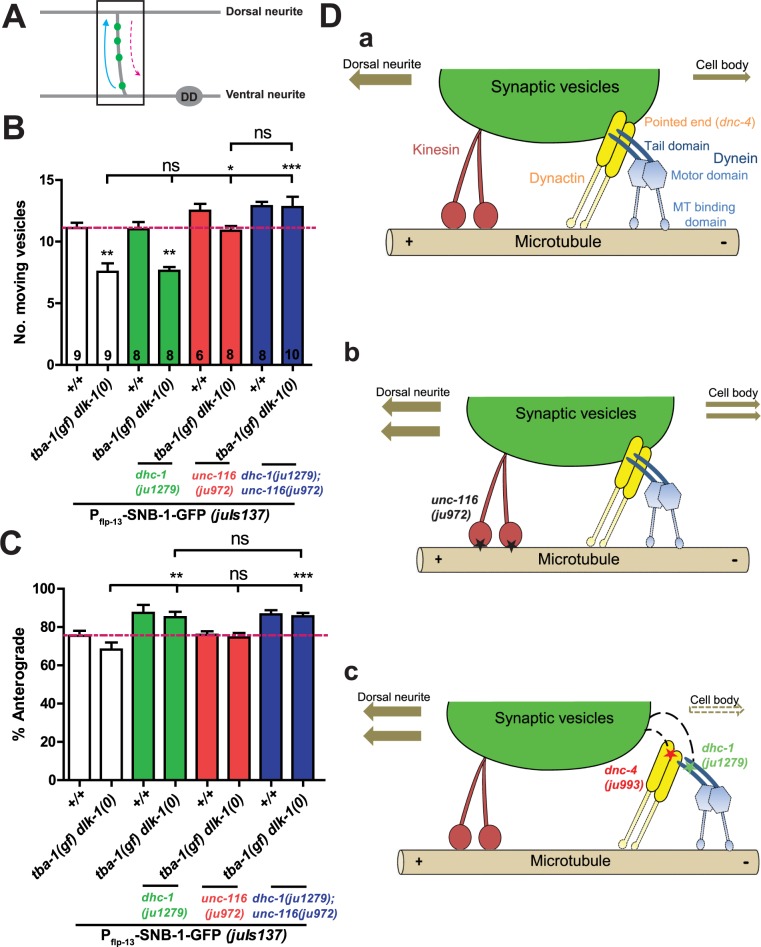
*dhc-1(ju1279)* enhances anterograde transport during synapse remodeling. (A) Schematic of imaging region (black box) in the DD neuron. SVs move in both the anterograde (blue solid arrow) and retrograde directions (pink dotted arrow) during remodeling. (B, C) Quantification of: (B) number of mobile vesicles, (C) their direction of movement during remodeling for various genotypes. Data are mean ± SEM; n = no. of animals (shown on (B)). Statistics: One-way ANOVA followed by Tukey’s posttest;*p<0.05, ***p<0.001, **p<0.01, n.s.-not significant (D Model of bidirectional cargo transport during DD neuron synapse remodeling. a) In wild type animals, kinesin (red) and the dynein (blue)-dynactin (yellow) complex transport SVs in both the anterograde and retrograde directions, with more SVs moving towards the dorsal neurite (anterograde). b) *unc-116(ju972)* (black stars) modifies the MT binding domain of kinesin to enhance both anterograde and retrograde SV transport. c) Almost all SVs move in the anterograde direction in *dhc-1(ju1279)* (green star) and *dnc-4(ju993)* (red star) animals, possibly due to a disruption in the interaction between the dynein-dynactin complex and SVs.

Since the *ju1279* allele affects the tail domain of DHC-1, which is structurally adjacent to the cargo binding domain of the dynein motor complex [30], we wondered whether SV transport was altered in the mutant animals. We assayed SV transport along the commissures of DD neurons during synapse remodeling in wild type and mutant animals using 4-dimensional (4-D) imaging (S1 Movie, Fig 3A). In wild type animals, most SVs moved in the anterograde direction, i.e., away from the cell body and towards their new location in the dorsal neurite (Fig 3C). This proportion of anterogradely moving SVs was maintained in *tba-1(gf) dlk-1(0)* animals (Fig 3C), albeit with a strong reduction in the total number of mobile SVs (Fig 3B) [11]. Addition of *dhc-1(ju1279)* did not change mobile SV numbers; instead we observed a significant increase in the proportion of SVs moving anterogradely in both *dhc-1(ju1279)* single and *dhc-1(ju1279) tba-1(gf) dlk-1(0)* triple mutant animals (Fig 3B and 3C). We also imaged SV transport in *dhc-1(js319)* animals during DD remodeling, and did not find any increase in anterogradely moving SVs. Together, these data indicate that an increase in anterogradely moving SVs in *dhc-1(ju1279) tba-1(gf) dlk-1(0)* triple mutant animals ameliorates a reduction in the total number of SVs reaching the dorsal neurite to promote synapse formation during remodeling.

We previously reported two suppressors (*ju972* and *ju977*) to be novel alleles of the anterograde motor, Kinesin-1/UNC-116 [11]. *unc-116(ju972)* strongly suppressed defective remodeling in *tba-1(gf) dlk-1(0)* by increasing total SV transport during remodeling, without altering the proportion of anterogradely moving SVs (Fig 3B–3D) [11]. Kinesins and dynein are classically thought of being in a “tug-of-war” during bi-directional cargo transport, highlighting the interdependence of the two motors for axonal transport in either direction (Fig 3D) [33–35]. We then wanted to see whether modifying dynein function using *dhc-1(ju1279)* would have any effect on SV transport in *unc-116(ju972)* animals. In both wild type and *tba-1(gf) dlk-1(0)* backgrounds, *dhc-1(ju1279); unc-116(ju972)* double mutant animals displayed a significant increase in both total number of mobile SVs and the proportion of anterogradely moving SVs, resulting in a strong anterograde bias in SV transport (Fig 3B and 3C). These observations led us to hypothesize that *dhc-1(ju1279)* likely weakens the interaction between the dynein complex and SVs, shifting the balance of bidirectional cargo transport in the anterograde direction (Fig 3D).

### The kinase TTBK-3 acts cell autonomously in DD neuron remodeling

**Fig 4:**
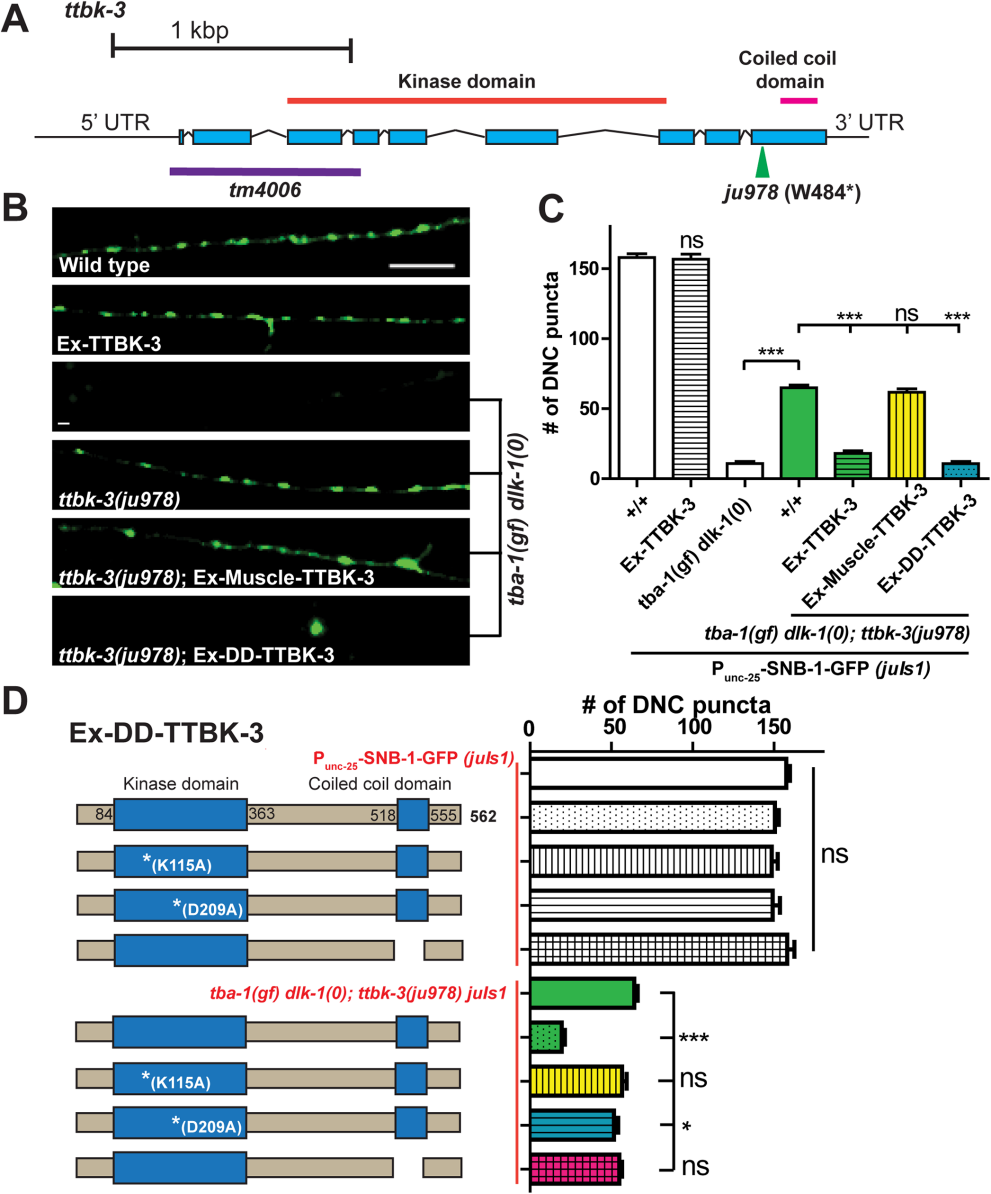
Kinase activity of *ttbk-3* is required for suppressing *tba-1(gf) dlk-1(0)*. (A) Gene structure of *ttbk-3*, with *ju978* and the deletion allele *tm4006* marked. (B) Representative images of DD neuron synapses along the DNC in adult animals imaged using *P*_*unc-25*_-SNB-1-GFP (*juIs1*). Ex-Muscle-TTBK-3 and Ex-DDneuron-TTBK-3 denotes extrachromosomal copies of wild type TTBK-3 expressed under *myo-3* (muscle) and *flp-13* (DD neuron) promoters, respectively. Scale bar: 10 μm. (C) Quantification of synaptic puncta in the DNC (*P*_*unc-25*_-SNB-1::GFP (*juIs1*)) of adult animals. Ex-TTBK-3 denotes extrachromosomal copies of wild type TTBK-3 expressed under its own promoter. Data are mean ± SEM; n>10 animals per genotype. Statistics: One-Way ANOVA followed by Tukey’s posttest; ***p<0.001, ns- not significant. (D) Quantification of synaptic puncta in the DNC (*P*_*unc-25*_-SNB-1::GFP (*juIs1*)) of adult animals. TTBK-3 contains a kinase domain and a C-terminal coiled-coil domain. Loss of either kinase domain activity (using kinase dead K115A and D209A mutants), or the coiled-coil domain in extrachromosomal copies of TTBK-3 (P_*flp-13*_-TTBK-3), result in a failure to rescue *tba-1(gf) dlk-1(0); ttbk-3(ju978) juIs1* animals. Data are mean ± SEM; n>8 animals per genotype. Statistics: One-Way ANOVA followed by Tukey’s posttest;*p<0.05, ***p<0.001, ns- not significant.

We mapped another suppressor mutation of *tba-1(gf) dlk-1(0)*, *ju978*, to the kinase *ttbk-3* (F32B6.10) (S1 Table and Fig 4A). *ttbk-3(tm4006)*, a deletion allele that removes the N-terminus and part of the kinase domain, also suppressed the synapse remodeling defects of *tba-1(gf) dlk-1(0)* (S3 Fig). Single mutants of *ju978* or *tm4006* were superficially wild type with no synapse formation or remodeling defects (Fig 4A–4C and S3 Fig). We verified that *ttbk-3(ju978)* was causative for suppression of *tba-1(gf) dlk-1(0)* by overexpressing wild type TTBK-3 in *tba-1(gf) dlk-1(0); ttbk-3(ju978)* animals, and observed a block in synapse remodeling in the transgenic animals (Fig 4B and 4C). These results indicate that loss of *ttbk-3* specifically restores synapse remodeling in *tba-1(gf) dlk-1(0)* animals.

TTBK-3 belongs to a large expansion of the Casein-kinase (CK1) superfamily in *C*. *elegans*, sharing 32% identity in the kinase domain to human tau-tubulin kinases (TTBK1 and TTBK2) [36]. Tau-tubulin kinases were first identified by their ability to phosphorylate the MT-associated protein tau and tubulin; mammalian TTBK1 is highly enriched in the nervous system, while TTBK2 is more broadly expressed [37, 38]. *ju978* generated a STOP codon in the C-terminal end of TTBK-3, which could result in a truncated protein with an intact kinase domain that lacked a coiled-coil domain further downstream (Fig 4A and 4D). Since *ttbk-3(ju978)* suppressed *tba-1(gf) dlk-1(0)* to a similar extent as *ttbk-3(tm4006)*, we asked whether the catalytic activity of *ttbk-3* was required for synapse remodeling. Overexpressing kinase-dead versions of TTBK-3 (K115A or D209A) [39] failed to rescue *tba-1(gf) dlk-1(0); ttbk-3(ju978)* (Fig 4D). We also obtained similar results using a mutant TTBK-3 lacking the C-terminal coiled-coil domain (Fig 4D). These results suggest that both the catalytic activity of the kinase domain and the coiled-coil domain are likely required for TTBK-3 function in synapse remodeling.

As reported by a recent study, expression of *ttbk-3* was weak and extremely variable [40] and we were not able to reliably examine its neuronal expression pattern using extrachromosomal arrays of GFP driven by the endogenous *ttbk-3* promoter. Expression of GFP tagged TTBK-3 in D motor neurons (*P*_*unc-25*_-TTBK-3-GFP) showed both diffuse and punctate GFP accumulation in the cell body and neurites during the L3 and L4 developmental stages, with a more diffuse distribution of GFP observed in adult animals (S3 Fig). GFP expression was almost undetectable outside the cell body during the period of DD synapse remodeling in L1 and L2 animals. To test if TTBK-3 acted cell autonomously in the DD neurons to regulate remodeling, we overexpressed TTBK-3 under a DD neuron specific promoter (P_*flp-13*_), which rescued the suppression of defective remodeling by *ttbk-3(ju978)* to a similar degree as full-length *ttbk-3*. As a control, expression of TTBK-3 from a muscle specific promoter (*myo-3*) failed to do so (Fig 4B and 4C), supporting the conclusion that TTBK-3 is required in DD neurons.

### TTBK-3 modulates nascent synapse stability after DD remodeling

Next, we focused on how *ttbk-3* could regulate synapse remodeling. Tau is a MAP that plays important roles in axonal transport, MT dynamics and neurite outgrowth during development, and is misregulated in neurodegenerative disease [41]. We tested a null mutation of the *C*. *elegans* homolog of tau, *ptl-1*, and found that *ptl-1(0)* did not have any effect on synapse formation or remodeling in wild type animals, and also failed to suppress the synapse remodeling defects of *tba-1(gf) dlk-1(0)* animals (S3 Fig). We also tested a null allele of *ttbk-7* (R90.1), the closest homolog of mammalian TTBK1/2 (65% sequence identity), which also failed to suppress *tba-1(gf) dlk-1(0)* (S3 Fig). These observations suggest *ttbk-3* likely acts through mechanisms independent of regulation of Tau.

**Fig 5:**
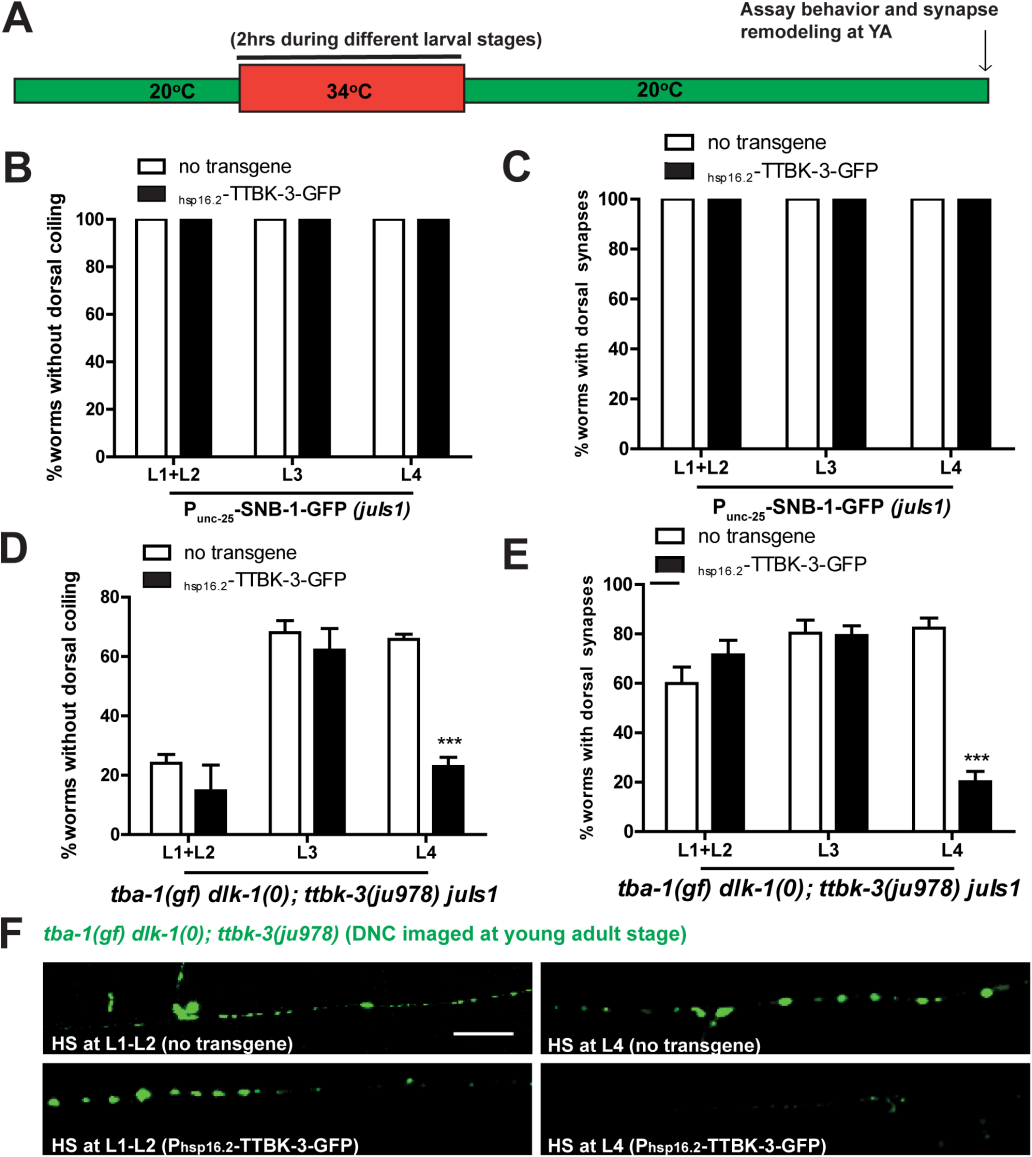
TTBK-3 is required for synapse maintenance during remodeling. (A) Schematic of heat shock assay, where animals are maintained at 20^°^C, undergo a 2hr heat shock during the developmental stage being tested, after which they are returned to 20^°^C and assayed at young adult stage. (B-E) Quantification of % animals with normal behavior (B, D) and synapse remodeling (C, E) following heat shock at L1-L4 stages. Data collected from 3 independent biological replicates, with n>10 animals each, and presented as mean ± SEM. Statistics- 2-Way ANOVA followed by Bonferroni posttest; ***p<0.001. (F) Representative images of DD neuron synapses along the DNC in adult animals imaged using *P*_*unc-25*_-SNB-1-GFP (*juIs1*). Scale bar: 10 μm.

We next imaged MT dynamics and SV transport and found that neither changed in *tba-1(gf) dlk-1(0); ttbk-3(tm4006)* animals when compared to *tba-1(gf) dlk-1(0)* animals (S3 Fig). Taken together with the expression pattern in DD neurons, this data suggested that *ttbk-3* was not involved in the early stages of synapse formation during remodeling. We then assayed the temporal requirement of *ttbk-3* in synapse remodeling using GFP tagged TTBK-3 expressed under a heat-shock inducible promoter (*P*_*hsp-16*.*2*_-TTBK-3-GFP). Following heat-shock in young adult animals TTBK-3-GFP formed punctate aggregates in neurons, the pharynx, the intestinal lumen and posterior intestinal cells (S3 Fig). To assay *ttbk-3* requirement, we induced TTBK-3 expression at various larval stages, and observed behavioral and synapse remodeling phenotypes in the induced animals at adulthood (Fig 5A). Heat-shock treated wild type animals (with or without TTBK-3-GFP) did not coil dorsally and had normal dorsal neurite synapse formation (Fig 5B and 5C). On the other hand, heat shock treatment of L4 stage *tba-1(gf) dlk-1(0); ttbk-3(ju978)* animals expressing TTBK-3-GFP significantly reduced the suppression of both behavioral and synapse remodeling defects by *ttbk-3(ju978)*, when compared to non-transgenic animals undergoing the same heat shock treatment (Fig 5D and 5E). We observed no difference in the extent of suppression between transgenic and non- transgenic animals that were heat-shocked at any other developmental stage, suggesting that *ttbk-3* specifically played a role in synapse remodeling at the L4 stage, and was not required for the induction of synapse remodeling at the L1-L2 stage. We previously reported the presence of transient dorsal neurite synaptic puncta in *tba-1(gf) dlk-1(0)* animals from the L2-L4 stage, which were then eliminated as the animal achieved adulthood [11]. Indeed, the intensity of SNB-1-GFP puncta observed along the dorsal neurite at the L4 stage was higher in *ttbk-3(tm4006)* animals, both in the wild type and *tba-1(gf) dlk-1(0)* backgrounds (S4 Fig). This suggested the existence of a mechanism to regulate the stabilization and maintenance of new synaptic sites formed on the dorsal neurite during remodeling. Since adding back wild type TTBK-3 at the L4 stage removed dorsal neurite synapses in *tba-1(gf) dlk-1(0); ttbk-3(ju978)* animals, we concluded that TTBK-3 antagonizes the stability of nascent synapses that are formed during synapse remodeling.

## Discussion

Modifications to synapse architecture occur throughout the lifetime of an animal, either dependent or independent of large scale neurite rewiring [4–9]. In this study, we dissect the mechanisms underlying a *C*. *elegans* model of structural synaptic plasticity, where synapses are eliminated and re-assembled along different neurites of the same neuron during a developmentally defined time scale [9–12].

We had previously shown that *tba-1(gf) dlk-1(0)* animals failed to complete synapse remodeling because of enhanced MT stability in DD neurons [11]. To identify additional regulators of the remodeling process, we screened for mutants that reversed the synaptic and behavioral defects of *tba-1(gf) dlk-1(0)* animals following EMS mutagenesis. Since we performed a non-clonal screen for homozygous viable adult animals, we were not able to isolate suppressors that might cause either lethality or sterility. Of the viable suppressors that we characterized, more than half were missense or nonsense alleles of *tba-1* that either reverted *tba-1(gf)* function back to wild type or resulted in null mutation of *tba-1*. Multiple mutations in human α-tubulin have been implicated in neuronal disorders like lissencephaly and ALS, and their effects on MTs have only been studied using *in vitro* overexpression models [42–44]. All the missense *tba-1* alleles that we identified in this screen alter invariant residues adjacent to disease-linked mutations in the C-terminal H12, the GTP binding domain and the loop between H8 and S7 (annotated in S1 Fig) [42–44], highlighting the importance of these three regions for α-tubulin functionality *in vivo*.

The non-tubulin suppressor mutations displayed incomplete suppression of the synapse remodeling defects of *tba-1(gf) dlk-1(0)*. On their own, none of these alleles had any effect on synapse formation or animal behavior. This might result from multiple factors. First, as was the case with motor proteins and their adaptors (*unc-116*, *dhc-1* and *dnc-4* alleles), the screen identified novel mutations that subtly altered protein function in such a way that embryonic development was completed and the animals were viable. Next, loss of function alleles in *ttbk-3(ju978)* behaved superficially wild type, likely because of the presence of other redundant kinases, which might also explain the incomplete suppression on *tba-1(gf) dlk-1(0)*. Finally, any suppressors with strong behavioral deficits were likely missed since the design of our screen aimed to identify animals with wild type behavior first, and then assayed for restoration of DD synapse remodeling.

A bottleneck to successful DD synapse remodeling is the motor dependent transport of SVs from the ventral to the dorsal neurite. Consistently, a majority of the suppressors altered either anterograde or retrograde motor function to compensate for a reduction in SV transport in *tba-1(gf) dlk-1(0)*. We previously identified kinesin-1 mutations that increased motor motility, and here, we characterized dynein-dynactin mutations that could alter cargo binding, with both strategies increasing anterograde SV transport along the DD neuron commissure during remodeling. However, while there was an overall increase in bidirectional SV transport in *unc-116(ju972)* animals, *dhc-1(ju1279)* selectively increased anterograde SV transport. This likely reflects the distinct motor domains targeted by *ju972* and *ju1279* on kinesin and dynein, respectively, with MT-motor interactions affecting bidirectional transport and variations in motor-cargo binding biasing transport in the direction with more engaged motors. Our results thus highlight both the interdependence and the competition between anterograde and retrograde motors during bidirectional cargo transport *in vivo*. The combination of *unc-116(ju972)* and *dhc-1(ju1279)* had an additive effect on SV transport, with an increase in both the total and anterogradely moving vesicle pools. Interestingly, *dhc-1(ju1279); unc-116(ju972)* did not enhance the number of dorsal neurite synapses in *tba-1(gf) dlk-1(0)* compared to *unc-116(ju972)* alone (S4 Fig), indicating that the synapse formation defects caused by *tba-1(gf)* alone [14] could not be suppressed simply by an increase in the number of available SVs.

Studies from various experimental models and cultured neurons indicate that nascent synapse stability is dependent on the transport of sufficient numbers of SVs to the site of new synapse formation [45–47]. In *tba-1(gf) dlk-1(0)* animals, the lack of sufficient SVs at sites of new synapse formation on the dorsal neurite resulted in their destabilization and subsequent elimination at the L4 stage [11]. Here, we found that TTBK-3, a *C*. *elegans* member of the casein kinase superfamily, promotes synapse destabilization along the dorsal neurite, and loss of *ttbk-3* was sufficient to maintain newly formed synapses in *tba-1(gf) dlk-1(0)*. Synapse destabilization by TTBK-3 is likely phosphorylation dependent, and future studies will be required to identify potential substrates. The coiled-coil domain was also required for TTBK-3 function, leading us to speculate a possible regulatory role for the domain on kinase function. We previously showed that the initiation of dorsal neurite synapse formation was mediated by *dlk-1* at the L2 stage; however *ttbk-3* does not appear to play a role at this stage [11]. *ttbk-3* is also not involved in ventral neurite synapse elimination during DD neuron remodeling, which is complete by L3 stage [11–13]. These results demonstrate that developmental synapse remodeling is marked by distinct phases of synapse formation, elimination and maintenance, each under tight spatio-temporal control to achieve precise neuronal connectivity.

## Materials and methods

### *C*. *elegans* culture

Strains were maintained at 20^°^C on NGM plates unless noted otherwise [48]. Information on alleles and genotypes of strains is summarized in S2 Table.

### Plasmid and transgene generation

Plasmids were generated using Gateway technology (Invitrogen). DNA for *dnc-4* and *ttbk-3* constructs was amplified from purified genomic or cDNA by PCR using Phusion HF DNA polymerase (Finnzyme) (S4 Table), and subcloned into PCR8 entry vectors. Transgenic animals were generated by microinjection, following standard procedures [49], using plasmids of interest at various concentrations (listed in S3 Table) and Pgcy-8-GFP (80–90 ng/μl) or Pmyo-2-mCherry (2.5 ng/μl) as co-injection markers. A minimum of 2–3 transgenes were generated for each construct described in this study. For rescue experiments using *dhc-1*, *dnc-4* and *ttbk-3* constructs, the data from 3 transgenes was pooled in statistical analyses. A list of the plasmids used in this study and the transgenic arrays generated from them is listed in S3 Table. Primer information for the DNA clones generated in this study is listed in S4 Table.

### Fluorescent imaging of synapses

L4 animals were cultured at 20^°^C overnight, and day 1 adults were imaged using a Zeiss LSM 710 confocal microscope. Animals were anaesthetized in 0.6 mM levamisole on 2% agar pads for image acquisition. Z-stacks were generated from slices of 0.6 μm thickness. Images were processed using Zen lite software. Synaptic puncta number was quantified manually using a Zeiss Axioplan 2 microscope equipped with Chroma HQ filters.

### SNB-1::GFP trafficking

L2 stage animals (14–18 hrs post hatching when maintained at 20^°^C) were collected for analysis, and anesthetized using 30 mM muscimol on 10% agarose pads. 4-D imaging was performed using a Yokogawa CSU-W1 spinning disc confocal head placed on a Leica DMi8 confocal microscope equipped with a piezo Z stage for fast Z- acquisition controlled using MetaMorph (Molecular Devices). The entire DD commissure was visualized in 5–6 slices and images were collected for a total of 20 frames. The resulting movies were analyzed using Metamorph to generate kymographs for analysis of number and direction of movement of synaptic vesicles.

### EBP-2::GFP image acquisition and analysis

Animals were anaesthetized in 30mM muscimol on 10% agarose pads for image acquisition. Live imaging for monitoring EBP-2 dynamics was done using a Yokogawa CSU-W1 spinning disc confocal head placed on a Leica DMi8 confocal microscope controlled by MetaMorph. 200 single plane images were taken serially at an exposure time of 113ms with an interval of 230ms between each frame, and analyzed using Metamorph software (Molecular Devices) to generate kymographs for analysis.

### Analysis of mutants from *tba-1(gf) dlk-1(0)* suppressor screen

*tba-1(gf) dlk-1(0)* animals were mutagenized using Ethyl Methane Sulphonate (EMS) following standard procedures [48]. F2 animals with improved locomotion were selected as putative suppressors in a non-clonal screen. Several suppressors were determined to be intragenic loss of function mutations in *tba-1(gf)* by Sanger sequencing the genic region of *tba-1* for any additional mutations. Two of the extragenic suppressors, *ju972* and *ju977*, were determined to be extragenic and mapped to the gene *unc-116* following whole genome sequence analysis by MAQGene [50]. The results of whole genome sequencing of the remaining suppressors were analyzed using a Galaxy (https://usegalaxy.org/) workflow, and the causative mutations were identified by linkage analysis of the suppression to the SNPs identified in the whole sequence analysis.

### Heat shock induced expression of *ttbk-3*

Transgenic animals expressing *P*_*hsp-16*.*2*_TTBK-3-GFP; *P*_*myo-2*_ mCherry in the wild type and *tba-1(gf) dlk-1(0); ttbk-3(0)* backgrounds were selected by positive pharyngeal mCherry expression. L1, L2, L3, L4 and young adult animals were heat shocked at 34^°^C for 2 hours in an incubator. Heat shocked animals were maintained at 20^°^C after heat shock until they reached day 1 adulthood, when they were imaged using a Zeiss LSM 710 confocal microscope.

### Statistical analysis

Statistical analysis was performed using GraphPad Prism 5. Normal distribution of samples was tested using D'Agostino & Pearson omnibus normality test. Significance was determined using One way ANOVA followed by Tukey’s multiple comparison tests and two way ANOVA followed by Bonferroni posttests for multiple samples.

## Supporting information

S1 Table Suppressors of *tba-1(gf) dlk-1(0)*. (DOCX)

S2 Table Strains and genotypes used in this study. (DOCX)

S3 Table List of constructs used in this study. (DOCX)

S4 Table List of cloning primers used in this study. (DOCX)

S1 Movie 4-D imaging of synaptic vesicle transport along a DD commissure. Synaptic vesicles move bidirectionally along the DD commissure towards the ventral and dorsal neurites.
(WMV)

S1 Fig (A) Gene structure of *tba-1*, with *tba-1(ju89gf)* and all the intragenic suppressors listed. Sequence alignment of parts of *C*.*elegans*, *H*. *sapiens* and *M*. *musculus* homologs of TBA-1. Sequence conservation of *ju89*, *ju962*, *ju965*, *ju973* and *ju987* is shown, as well as the location of various helices (H) and beta sheets (B). Also annotated are mutations linked to ALS (in green) and lissencephaly (in blue) disease phenotypes in patient samples. (B) Gene structure of *tbb-2*, with *ju1535* and reference allele *gk129* marked. Sequence alignment of parts of *C*.*elegans*, *H*. *sapiens* and *M*. *musculus* homologs of TBB-2, highlighting the conserved Proline that is altered in *ju1535*, which lies between H9 and H10 helices of *tbb-2*. (C) Structure prediction of *C*. *elegans* TBA-1 (based on PDB#4i4tc) modeled on SWISS-MODEL and rendered using PyMOL, with *ju89* (G414R) and the various missense mutations identified during the suppressor screen are also marked. (D) Structure prediction of *C*. *elegans* TBA-1 and TBB-2 (based on PDB# 1JFF) modeled on SWISS-MODEL and rendered using PyMOL, with the position of the internal and external MT surface, and the positions of *ju89* and *ju1535* highlighted.
(PDF)

S2 Fig (A) Alignment of sequences of dynein heavy chains from *C*.*elegans*, *H*. *sapiens* and *M*. *musculus*, surrounding the conserved Proline residue that is mutated in *ju1279*. (B) Bright field images of various *dhc-1* alleles (homozygous *ju1279* and *js319*, and heterozygous *ju1279/js319*) cultured at 25^°^C. (C, D) Representative kymographs of EBP-2 movement in the adult VNC of the respective genotypes. Red lines indicate EBP-2 comets moving in the anterograde direction. (E, F) Quantification of number of EBP-2 comets (D) and their direction of movement (E) for various genotypes. Data are mean ± SEM; n = number of animals (shown on (E)). Statistics: One-way ANOVA followed by Tukey’s posttest; ***p<0.001, n.s.-not significant. (G) Bright field images of various *dnc-4* alleles (homozygous *ju933* and *or633*, and heterozygous *ju933/or633*) cultured at 25^°^C.
(PDF)

S3 Fig (A) Representative images of DD synapses along the DNC in adult animals using P_*flp-13*_-SNB-1-GFP (*juIs137*). Scale bar: 10 μm. (B) Quantification of synaptic puncta in the DNC of adult animals. Data are mean ± SEM; n>10 animals per genotype. Statistics: One-Way ANOVA followed by Tukey’s posttest; ***p<0.001, ns- not significant. (C) Representative images of synaptic puncta along the DNC imaged using *P*_*unc-25*_-SNB-1-GFP (*juIs1*). Scale bar: 10 μm. (D) Representative images of TTBK-3-GFP expression in the GABAergic D motor neurons (driven by the *unc-25* promoter) in L3, L4 and adult animals. (E) Quantification of number of EBP-2 comets for various genotypes. Data are mean ± SEM; Statistics: One-way ANOVA followed by Tukey’s posttest; ***p<0.001, n.s.-not significant. (F) Representative images of young adult wild type animals carrying the P_*hsp-16*.*2*_-TTBK-3-GFP transgene, 4 hours after a 2hr heat shock period. Punctate structures are seen in the pharynx, some head neurons, the intestinal lumen, CAN neuron and a posterior intestinal cell. Scale bar: 10 μm.
(PDF)

S4 Fig (A, B) Quantification of SNB-1::GFP intensity (P_*flp-13*_-SNB-1-GFP (*juIs137*)) in the DNC of L4 animals, anaesthetized using 30mM muscimol. Data are mean ± SEM; n = 10 animals per genotype. Statistics: Mann-Whitney test, p-values are displayed on graph. (C) Quantification of synaptic puncta in the DNC of adult animals. Data are mean ± SEM; n>10 animals per genotype. Statistics: One-Way ANOVA followed by Tukey’s posttest; ***p<0.001, ns- not significant.
(PDF)
