# Supplementary material for: Differential regulation of polarized synaptic vesicle trafficking and synapse stability in neural circuit rewiring in Caenorhabditis elegans
Source: PLoS Genet. 2017 Jun 21;13(6):e1006844. doi: 10.1371/journal.pgen.1006844 (PMC5500376; doi:10.1371/journal.pgen.1006844)
Supplement: S1 Table — (DOCX) [file pgen.1006844.s001.docx]

**Supplementary Table 1: Suppressors of *tba-1(gf) dlk-1(0)***

| **Allele** | **Gene** | **Mutation**  **Amino acid (Nucleotide)** | **Function** |
| --- | --- | --- | --- |
| ***ju962*** | ***tba-1*** | **S285F (C854T)** | **MT architecture** |
| ***ju964*** | ***tba-1*** | **K278*(A837T)** | **MT architecture** |
| ***ju965*** | ***tba-1*** | **S138L(C413T)** | **MT architecture** |
| ***ju966*** | ***tba-1*** | **Q41*(C121T)** | **MT architecture** |
| ***ju973*** | ***tba-1*** | **L426F(C1371T)** | **MT architecture** |
| ***ju975*** | ***tba-1*** | **Y183*(C599A)** | **MT architecture** |
| ***ju980*** | ***tba-1*** | **M1I(G3A)** | **MT architecture** |
| ***ju987*** | ***tba-1*** | **A419T(G1350A)** | **MT architecture** |
| ***ju1535*** | ***tbb-2*** | **P305S(C1062T)** | **MT architecture** |
| ***ju972*** | ***unc-116*** | **G274R(G1400A)** | **Transport** |
| ***ju977*** | ***unc-116*** | **E432K(G1921A)** | **Transport** |
| ***ju1279*** | ***dhc-1*** | **P262L(C832T)** | **Transport** |
| ***ju993*** | ***dnc-4*** | **S368F(G841A)** | **Transport** |
| ***ju978*** | ***ttbk-3*** | **W484*(G2440A)** | **Kinase** |
| ***ju982*** | ***ifp-1*** | **L363F(C1087T)** | **Cytoskeleton** |
| ***ju963*** | ***ifp-1*** | **P443S(C1327T)** | **Cytoskeleton** |
