# Supplementary material for: Differential regulation of polarized synaptic vesicle trafficking and synapse stability in neural circuit rewiring in Caenorhabditis elegans
Source: PLoS Genet. 2017 Jun 21;13(6):e1006844. doi: 10.1371/journal.pgen.1006844 (PMC5500376; doi:10.1371/journal.pgen.1006844)
Supplement: S2 Table — (DOCX) [file pgen.1006844.s002.docx]

**Supplementary Table 2: Strains and Genotypes used in this study**

| **Strain** | **Genotype** | **Allele or Transgene** |
| --- | --- | --- |
| CZ333 | *juIs1 IV* | *juIs1* [*Punc-25-SNB-1::GFP; lin-15(+)*] |
| CZ2569 | *tba-1(ju89) I; juIs1 IV* | *ju89*: Gly414Arg |
| CZ15940 | *dlk-1(tm4024) I; juIs1 IV* | *tm4024*: 460 bp deletion |
| CZ12121 | *tba-1(ju89)dlk-1(tm4024) I; juIs1 IV* |  |
| CZ2060 | *juIs137 II* | *juIs137* [*Pflp-13-_SNB_-1::GFP; lin-15(+)*] |
| CZ18652 | *dlk-1(tm4024) I; juIs137 II* |  |
| CZ2411 | *tba-1(ju89) I; juIs137 II* |  |
| CZ12177 | *sem-4(n1378) tba-1(ju89) tba-1(ju962) dlk-1(tm4024) I; juIs1 IV* | *ju962*: Ser285Phe |
| CZ12179 | *sem-4(n1378) tba-1(ju89) tba-1(ju964) dlk-1(tm4024) I; juIs1 IV* | *ju964*: Lys278* |
| CZ12180 | *sem-4(n1378) tba-1(ju89) tba-1(ju965) dlk-1(tm4024) I; juIs1 IV* | *ju965*: Ser138Leu |
| CZ12181 | *sem-4(n1378) tba-1(ju89) tba-1(ju966) dlk-1(tm4024) I; juIs1 IV* | *ju966*: Gln41* |
| CZ12345 | *tba-1(ju89) tba-1(ju973) dlk-1(tm4024) I; juIs1 IV* | *ju973*: Leu426Phe |
| CZ12363 | *tba-1(ju89) tba-1(ju975) dlk-1(tm4024) I; juIs1 IV* | *ju975*: Tyr183* |
| CZ12368 | *tba-1(ju89) tba-1(ju980) dlk-1(tm4024) I; juIs1 IV* | *ju980*: Met1Iso |
| CZ12375 | *tba-1(ju89) tba-1(ju987) dlk-1(tm4024) I; juIs1 IV* | *ju987*: Ala419Thr |
| C21593 | *tba-1(ju89) dlk-1(tm4024) I; juIs1 IV; ifp-1(ju982) X* | *ju982*: Leu363Phe |
| CZ21594 | *tba-1(ju89) dlk-1(tm4024) I; juIs137 II; ifp-1(ju982) X* |  |
| CZ21596 | *dlk-1(tm4024) I; juIs137 II; ifp-1(ju982) X* |  |
| CZ21595 | *tba-1(ju89) I; juIs137 II; ifp-1(ju982) X* |  |
| CZ21597 | *juIs137 II; ifp-1(ju982) X* |  |
| CZ25360 | *tbb-2(ju1535) III; juIs1 IV* | *ju1535*: Pro305Ser |
| CZ25355 | *tba-1(ju89) I; tbb-2(ju1535) III; juIs1 IV* |  |
| CZ25359 | *tba-1(ju89) dlk-1(tm4024) I; tbb-2(ju1535) III; juIs1 IV* |  |
| CZ25565 | *tba-1(ju89) dlk-1(tm4024) I; tbb-2(gk129) III; juIs1 IV* | *gk129*: 766 bp deletion |
| CZ25564 | *tbb-2(gk129) III; juIs1 IV* |  |
| CZ25571 | *juIs1 IV; juEx* | *juEx7692* [Ptbb-2-TBB-2; gcy-8-GFP] |
| CZ25574 | *tba-1(ju89) dlk-1(tm4024) I; tbb-2(ju1535) III; juIs1 IV; juEx* | *juEx7695* [Ptbb-2-TBB-2; gcy-8-GFP] |
| CZ21346 | *dhc-1(ju1279) tba-1(ju89) dlk-1(tm4024) I; juIs1 IV; ifp-1(ju963) X* | *ju963*: Pro443Ser  *ju1279*: Pro262Leu |
| CZ21819 | *tba-1(ju89) dlk-1(tm4024) I; juIs1 IV; ifp-1(ju963) X* |  |
| CZ15115 | *tba-1(ju89) dlk-1(tm4024) I; unc-116(ju977) III; juIs1 IV* | *ju977*: Glu432Lys |
| CZ23620 | *tba-1(ju89) dlk-1(tm4024) I; dnc-4(ju993) juIs1 IV* | *ju993*: Val229Iso |
| CZ22433 | *tba-1(ju89) dlk-1(tm4024) I; ttbk-3(ju978) juIs1 IV* | *ju978*: Trp484* |
| CZ22439 | *dhc-1(ju1279) I; juIs137 II* |  |
| CZ24719 | *dhc-1(js319) I; juIs137 II* | *js319*: Splice site mutation |
| CZ16989 | *tba-1(ju89)dlk-1(tm4024) I; juIs137 II* |  |
| CZ22440 | *dhc-1(ju1279) tba-1(ju89) dlk-1(tm4024) I; juIs137 II* |  |
| CZ16994 | *tba-1(ju89) dlk-1(tm4024) I;unc-116(ju972) III; juIs1 IV* | *ju972*: Gly274Arg |
| CZ16633 | *unc-116(ju972) III; juIs1 IV* |  |
| CZ16991 | *tba-1(ju89) I; unc-116(ju972) III; juIs1 IV* |  |
| CZ17360 | *dlk-1(tm4024) I; unc-116(ju972) III; juIs1 IV* |  |
| CZ16631 | *tba-1(ju89)dlk-1(tm4024) I* |  |
| CZ17824 | *juEx5317* | *juEx5317* [*Punc-25-EBP-2::GFP; Pgcy-8-GFP*] |
| CZ20218 | *tba-1(ju89)dlk-1(tm4024) I; juEx5317* |  |
| CZ23623 | *dhc-1(ju1279) I; juEx5317* |  |
| CZ23624 | *dhc-1(ju1279) tba-1(ju89)dlk-1(tm4024) I; juEx5317* |  |
| CZ23625 | *ttbk-3(tm4006) IV; juEx5317* | *tm4006*: 786 bp deletion + 21 bp insertion |
| CZ23626 | *tba-1(ju89) dlk-1(tm4024) I; ttbk-3(tm4006) IV; juEx5317* |  |
| CZ24382 | *juIs137 II; juEx7441* | *juEx7441* [*Fosmid- WRM0639aB10; Pgcy-8-GFP*] |
| CZ24385 | *dhc-1(ju1279) tba-1(ju89) dlk-1(tm4024) I; juIs137 II; juEx7444* | *juEx7444*  [*Fosmid- WRM0639aB10; Pgcy-8-GFP*] |
| CZ17362 | *juIs137 II; unc-116(ju972) III* |  |
|  | *tba-1(ju89) dlk-1(tm4024) I; juIs137 II; unc-116(ju972) III* |  |
| CZ24580 | *dhc-1(ju1279) I; juIs137 II; unc-116(ju972) III* |  |
| CZ24581 | *dhc-1(ju1279) I; tba-1(ju89) dlk-1(tm4024) I; juIs137 II; unc-116(ju972) III* |  |
| CZ22741 | *dnc-4(ju993) juIs1 IV* |  |
| CZ23893 | *dnc-4(ju993) juIs1 IV; juEx7288* | *juEx7288*  [*Pdnc-4-DNC-4; Pgcy-8-GFP*] |
| CZ23896 | *tba-1(ju89) dlk-1(tm4024) I; dnc-4(ju993) juIs1 IV; juEx7291* | *juEx7291*  [*Pdnc-4-DNC-4; Pgcy-8-GFP*] |
| EU1506 | *dnc-4(or633) IV* | *or633*: Glu42Lys |
| CZ23901 | *tba-1(ju89) dlk-1(tm4024) I; juIs137 II; dnc-4(or633) IV* |  |
| CZ22866 | *juIs1 IV; juEx6991* | *juEx6991*  [*Pttbk-3-TTBK-3; Pgcy-8-GFP*] |
| CZ22869 | *tba-1(ju89) dlk-1(tm4024) I; ttbk-3(ju978) juIs1 IV; juEx6994* | *juEx6994*  [*Pttbk-3-TTBK-3; Pgcy-8-GFP*] |
| CZ23362 | *tba-1(ju89) dlk-1(tm4024) I; ttbk-3(ju978) juIs1 IV; juEx* | *juEx7141*  [*Pmyo-3-TTBK-3; Pgcy-8-GFP*] |
| CZ23356 | *tba-1(ju89) dlk-1(tm4024) I; ttbk-3(ju978) juIs1 IV; juEx* | *juEx7135*  [*Pflp-13-TTBK-3; Pgcy-8-GFP*] |
| CZ24387 | *juIs1 IV; juEx7447* | *juEx7447*  [*Pflp-13-TTBK-3(cDNA full length); Pgcy-8-GFP*] |
| CZ24394 | *juIs1 IV; juEx7453* | *juEx7453*  [*Pflp-13-TTBK-3(cDNA (∆CC)); Pgcy-8-GFP*] |
| CZ23627 | *juIs1 IV; juEx7235* | *juEx7235*  [*Pflp-13-TTBK-3(cDNA (K115A)); Pgcy-8-GFP*] |
| CZ23628 | *juIs1 IV; juEx7236* | *juEx7236*  [*Pflp-13-TTBK-3(cDNA (D209A); Pgcy-8-GFP*] |
| CZ24391 | *tba-1(ju89) dlk-1(tm4024) I; ttbk-3(ju978) juIs1 IV; juEx7450* | *juEx7450*  [*Pflp-13-TTBK-3(cDNA full length); Pgcy-8-GFP*] |
| CZ24397 | *tba-1(ju89) dlk-1(tm4024) I; ttbk-3(ju978) juIs1 IV; juEx7456* | *juEx7456*  [*Pflp-13-TTBK-3(cDNA (∆CC)); Pgcy-8-GFP*] |
| CZ23629 | *tba-1(ju89) dlk-1(tm4024) I; ttbk-3(ju978) juIs1 IV; juEx7237* | *juEx7237*  [*Pflp-13-TTBK-3(cDNA (K115A)); Pgcy-8-GFP*] |
| CZ23632 | *tba-1(ju89) dlk-1(tm4024) I; ttbk-3(ju978) juIs1 IV; juEx7241* | *juEx7241*  [*Pflp-13-TTBK-3(cDNA (D209A)); Pgcy-8-GFP*] |
| CZ22965 | *juIs137 II; ttbk-3(tm4006) IV* |  |
| CZ23149 | *tba-1(ju89) dlk-1(tm4024); juIs137 II; ttbk-3(tm4006) IV* |  |
| CZ24720 | *tba-1(ju89) dlk-1(tm4024); juIs137 II; ttbk-7(tm4852) V* | *tm4852*: 1051 bp deletion + 9 bp insertion |
| CZ22962 | *tba-1(ju89) dlk-1(tm4024); ptl-1(ok621) III; juIs1 IV* | *ok621*: 1933 bp deletion |
| CZ24570 | *juIs1 IV; juEx7537* | *juEx7537*  [*Phsp16.2-TTBK-3-GFP; Pmyo-2-mCherry*] |
| CZ24574 | *tba-1(ju89) dlk-1(tm4024) I; ttbk-3(ju978) juIs1 IV; juEx7541* | *juEx7541*  [*Phsp16.2-TTBK-3-GFP; Pmyo-2-mCherry*] |
| CZ25487 | *juEx7680* | *juEx7680*  [*Punc-25-TTBK-3-GFP; Pgcy-8-GFP*] |
