# Supplementary material for: Differential regulation of polarized synaptic vesicle trafficking and synapse stability in neural circuit rewiring in Caenorhabditis elegans
Source: PLoS Genet. 2017 Jun 21;13(6):e1006844. doi: 10.1371/journal.pgen.1006844 (PMC5500376; doi:10.1371/journal.pgen.1006844)
Supplement: S3 Table — (DOCX) [file pgen.1006844.s003.docx]

**Supplementary Table 3: List of constructs used in this study**

| **Plasmid** | **Description** | **Transgenes generated** |
| --- | --- | --- |
| pCZGY3197 | tbb-2(2.4 kb promoter)-tbb-2 genomic DNA–tbb-2 3’UTR (156 bp) | *juEx7692-97* (Injected at 5 ng/μl) |
| pCZGY2332 | unc-25(2 kb promoter)-EBP-2 cDNA-mGFP-unc-54 3’UTR | *juEx5317, juEx5318* (Injected at 5 ng/μl) |
| pCZGY3198 | dnc-4(1.5 kb promoter)-dnc-4 genomic DNA–dnc-4 3’UTR (200 bp) | *juEx7288-93* (Injected at 20 ng/ μl) |
| pCZGY3199 | ttbk-3(600 bp promoter)-ttbk-3 genomic DNA –ttbk-3 3’UTR (97 bp) | *juEx6991-96* (Injected at 10 ng/ μl) |
| pCZGY3201 | flp-13(2.1 kb promoter)- ttbk-3 genomic DNA -unc-54 3’UTR | *juEx7132-37* (Injected at 20 ng/ μl) |
| pCZGY3202 | myo-3(2.4 kb promoter)- ttbk-3 genomic DNA -unc-54 3’UTR | *juEx7138-43* (Injected at 20 ng/ μl) |
| pCZGY3207 | flp-13(2.1 kb promoter)-ttbk-3 cDNA(full length)-unc-54 3’UTR | *juEx7447-52* (Injected at 1 ng/ μl) |
| pCZGY3208 | flp-13(2.1 kb promoter)-ttbk-3 cDNA(∆CC)-unc-54 3’UTR | *juEx7453-58* (Injected at 1 ng/ μl) |
| pCZGY3209 | flp-13(2.1 kb promoter)-ttbk-3 cDNA(K115A)-unc-54 3’UTR | *juEx7235;*  *juEx7237-39* (Injected at 20 ng/ μl) |
| pCZGY3210 | flp-13(2.1 kb promoter)-ttbk-3 cDNA(D209A)-unc-54 3’UTR | *juEx7236;*  *juEx7240-42* (Injected at 20 ng/ μl) |
| pCZGY3211 | hsp16.2(436 bp promoter)-ttbk-3genomic DNA-GFP-unc-54 3’UTR | *juEx7537-7542* (Injected at 50 ng/ μl) |
| pCZGY3212 | unc-25(1.2 kb promoter)-ttbk-3genomic DNA-GFP-unc-54 3’UTR | *juEx7680-7681*(Injected at 50 ng/μl) |
