# Supplementary material for: Differential regulation of polarized synaptic vesicle trafficking and synapse stability in neural circuit rewiring in Caenorhabditis elegans
Source: PLoS Genet. 2017 Jun 21;13(6):e1006844. doi: 10.1371/journal.pgen.1006844 (PMC5500376; doi:10.1371/journal.pgen.1006844)
Supplement: S4 Table — (DOCX) [file pgen.1006844.s004.docx]

**Supplementary Table 4: List of cloning primers used in this study**

| **Primer** | **Sequence** | **Description** |
| --- | --- | --- |
| YJ12226 | CGCAATAATGCACATTTGTGTG | Forward primer for amplifying TBB-2 genomic sequence to generate pCZGY3197 |
| YJ12227 | gatagcattcacttcactcagatgc | Reverse primer for amplifying TBB-2 genomic sequence to generate pCZGY3197 |
| YJ12228 | GCACGAATTTGGAAAACTCG | Forward primer for amplifying DNC-4 genomic sequence to generate pCZGY3198 |
| YJ12229 | TACTCCATCTTGCCAGAATATTG | Reverse primer for amplifying DNC-4 genomic sequence to generate pCZGY3198 |
| YJ12230 | GATGTAATGTTGAAGTGAGAGTAGCC | Forward primer for amplifying TTBK-3 genomic sequence to generate pCZGY3199 |
| YJ12231 | gcagctcagatattttttatgtgcc | Reverse primer for amplifying TTBK-3 genomic sequence to generate pCZGY3199 |
| YJ12232 | atggttgatGTAAGTGACAATGTTTTC | Forward primer for amplifying TTBK-3 genomic sequence (without promoter) to generate pCZGY3201-3202 and pCZGY3211-3212 |
| YJ12233 | ATGGTTGATAAAAACAAAAAGAATCTTCC | Forward primer for amplifying TTBK-3 cDNA sequence to generate pCZGY3207-3210 |
| YJ12234 | TTATTTTGGAGATGAATTGAAAGTTCC | Reverse primer for amplifying TTBK-3 cDNA sequence to generate pCZGY3207-3210 |
| YJ12235 | GTCGCTCTGAAGTATTTGACGACTTTCAATTCATCTCCAA | Forward primer for site directed mutagenesis to generate pCZGY3208 from pCZGY3207 |
| YJ12236 | TTGGAGATGAATTGAAAGTCGTCAAATACTTCAGAGCGAC | Reverse primer for site directed mutagenesis to generate pCZGY3208 from pCZGY3207 |
| YJ12237 | GATCAGTATTACGCTGTAGCAATAGAAGTTCGCCTTACCTC | Forward primer for site directed mutagenesis to generate pCZGY3209 from pCZGY3207 |
| YJ12238 | GAGGTAAGGCGAACTTCTATTGCTACAGCGTAATACTGATC | Reverse primer for site directed mutagenesis to generate pCZGY3209 from pCZGY3207 |
| YJ12239 | AAGGTTTCATTCATCGAGCTATAAAACCGGAAAACCTATTGA | Forward primer for site directed mutagenesis to generate pCZGY3210 from pCZGY3207 |
| YJ12240 | TCAATAGGTTTTCCGGTTTTATAGCTCGATGAATGAAACCTT | Reverse primer for site directed mutagenesis to generate pCZGY3210 from pCZGY3207 |
