# Supplementary figures and images for: Differential regulation of polarized synaptic vesicle trafficking and synapse stability in neural circuit rewiring in Caenorhabditis elegans
Source: PLoS Genet. 2017 Jun 21;13(6):e1006844. doi: 10.1371/journal.pgen.1006844 (PMC5500376; doi:10.1371/journal.pgen.1006844)

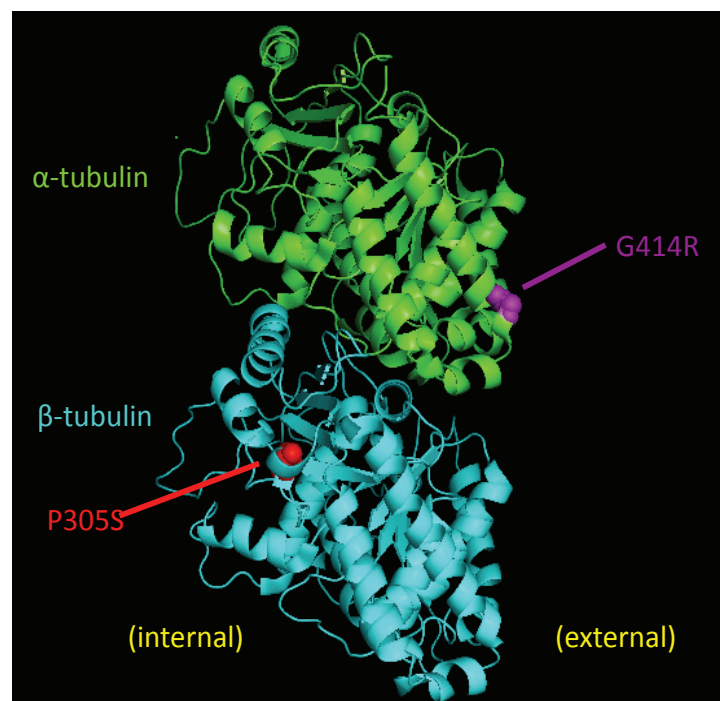

Supplement: S1 Fig — (A) Gene structure of tba-1, with tba-1(ju89gf) and all the intragenic suppressors listed. Sequence alignment of parts of C.elegans, H. sapiens and M. musculus homologs of TBA-1. Sequence conservation of ju89, ju962, ju965, ju973 and ju987 is shown, as well as the location of various helices (H) and beta sheets (B). Also annotated are mutations linked to ALS (in green) and lissencephaly (in blue) disease phenotypes in patient samples. (B) Gene structure of tbb-2, with ju1535 and reference allele gk129 marked. Sequence alignment of parts of C.elegans, H. sapiens and M. musculus homologs of TBB-2, highlighting the conserved Proline that is altered in ju1535, which lies between H9 and H10 helices of tbb-2. (C) Structure prediction of C. elegans TBA-1 (based on PDB#4i4tc) modeled on SWISS-MODEL and rendered using PyMOL, with ju89 (G414R) and the various missense mutations identified during the suppressor screen are also marked. (D) Structure prediction of C. elegans TBA-1 and TBB-2 (based on PDB# 1JFF) modeled on SWISS-MODEL and rendered using PyMOL, with the position of the internal and external MT surface, and the positions of ju89 and ju1535 highlighted. (PDF) [file pgen.1006844.s006.pdf]

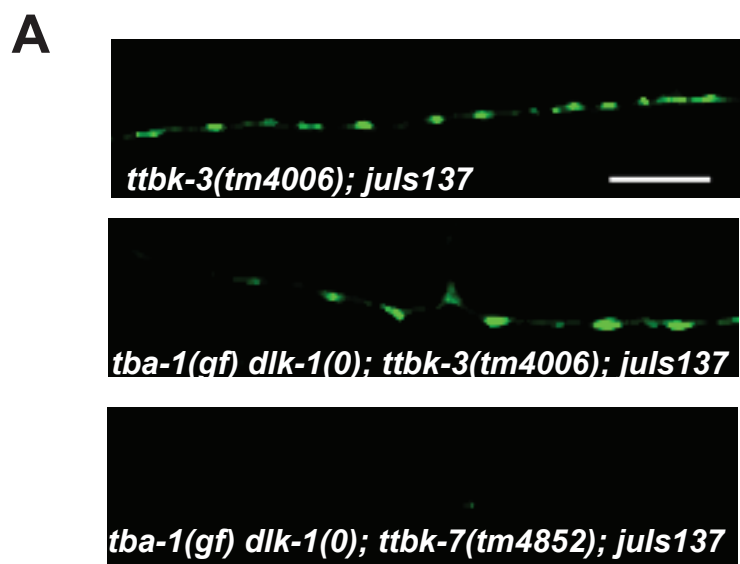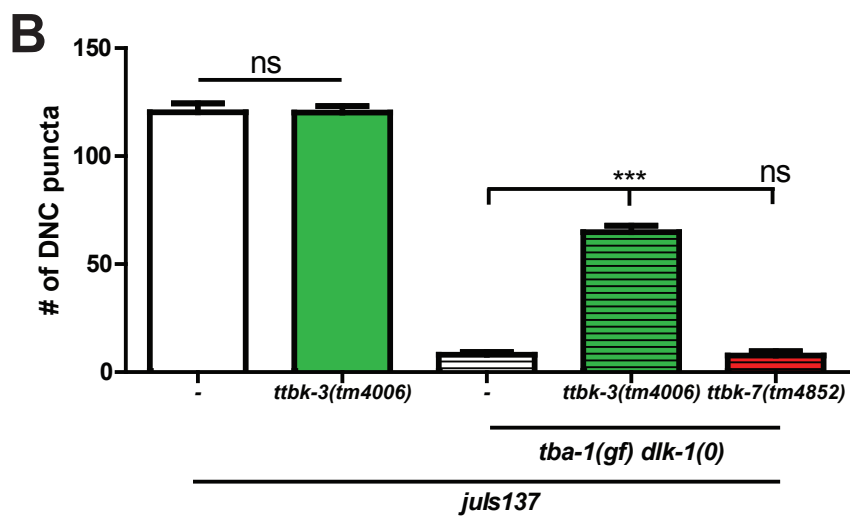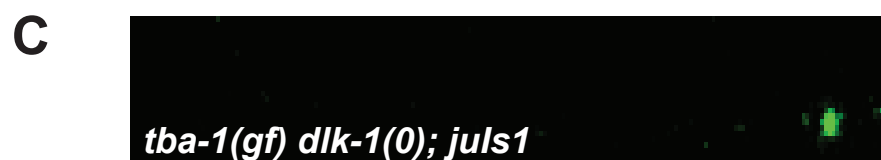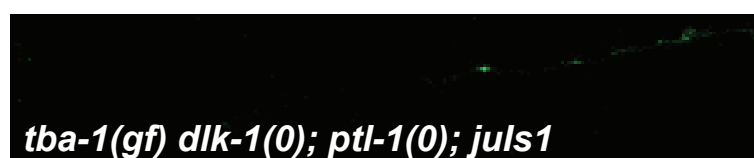

**D** Punc-25-TTBK-3-GFP (VNC)

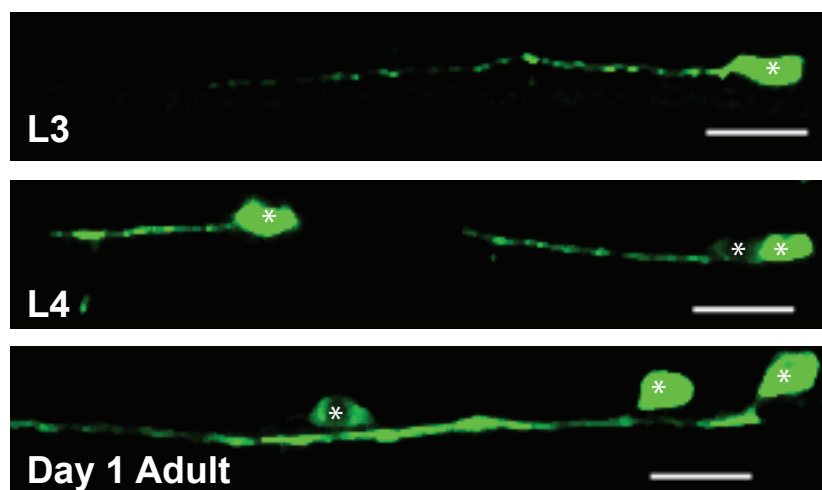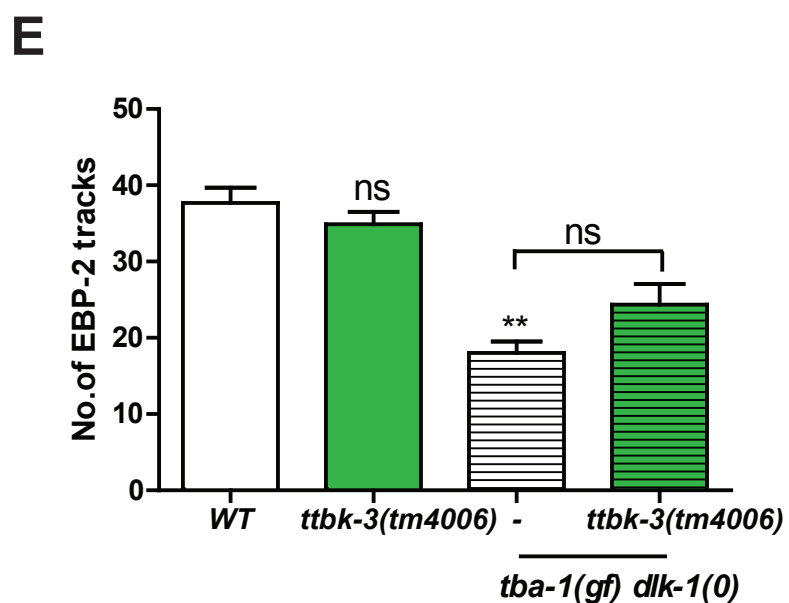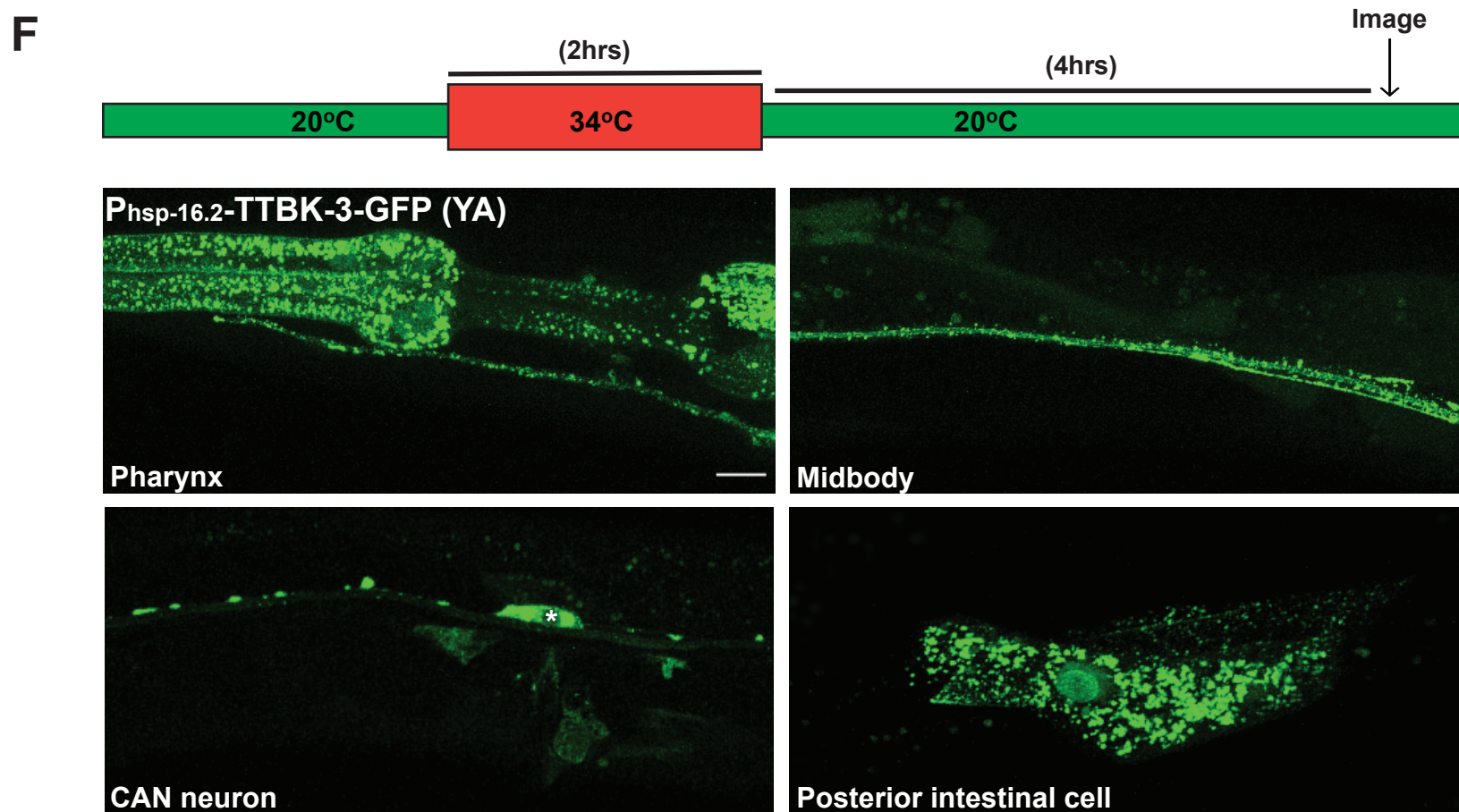

Supplement: S3 Fig — (A) Representative images of DD synapses along the DNC in adult animals using Pflp-13-SNB-1-GFP (juIs137). Scale bar: 10 μm. (B) Quantification of synaptic puncta in the DNC of adult animals. Data are mean ± SEM; n>10 animals per genotype. Statistics: One-Way ANOVA followed by Tukey’s posttest; ***p<0.001, ns- not significant. (C) Representative images of synaptic puncta along the DNC imaged using Punc-25-SNB-1-GFP (juIs1). Scale bar: 10 μm. (D) Representative images of TTBK-3-GFP expression in the GABAergic D motor neurons (driven by the unc-25 promoter) in L3, L4 and adult animals. (E) Quantification of number of EBP-2 comets for various genotypes. Data are mean ± SEM; Statistics: One-way ANOVA followed by Tukey’s posttest; ***p<0.001, n.s.-not significant. (F) Representative images of young adult wild type animals carrying the Phsp-16.2-TTBK-3-GFP transgene, 4 hours after a 2hr heat shock period. Punctate structures are seen in the pharynx, some head neurons, the intestinal lumen, CAN neuron and a posterior intestinal cell. Scale bar: 10 μm. (PDF) [file pgen.1006844.s008.pdf]

**A**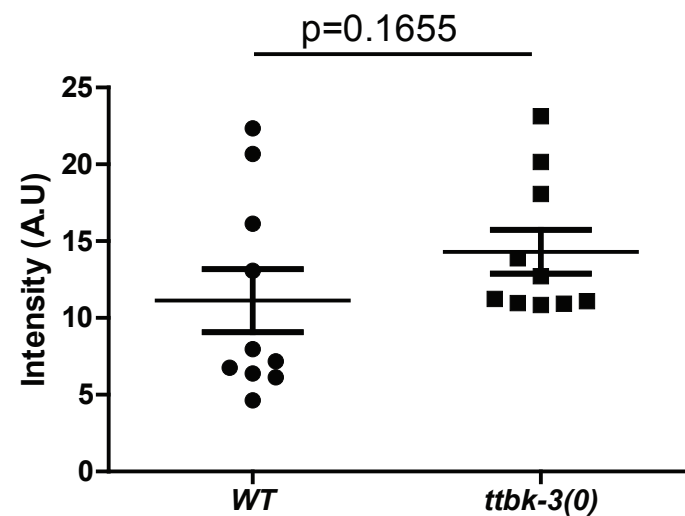**B**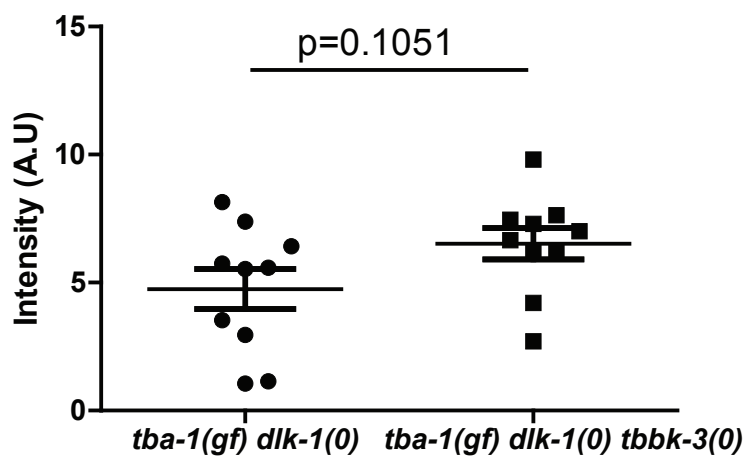**C**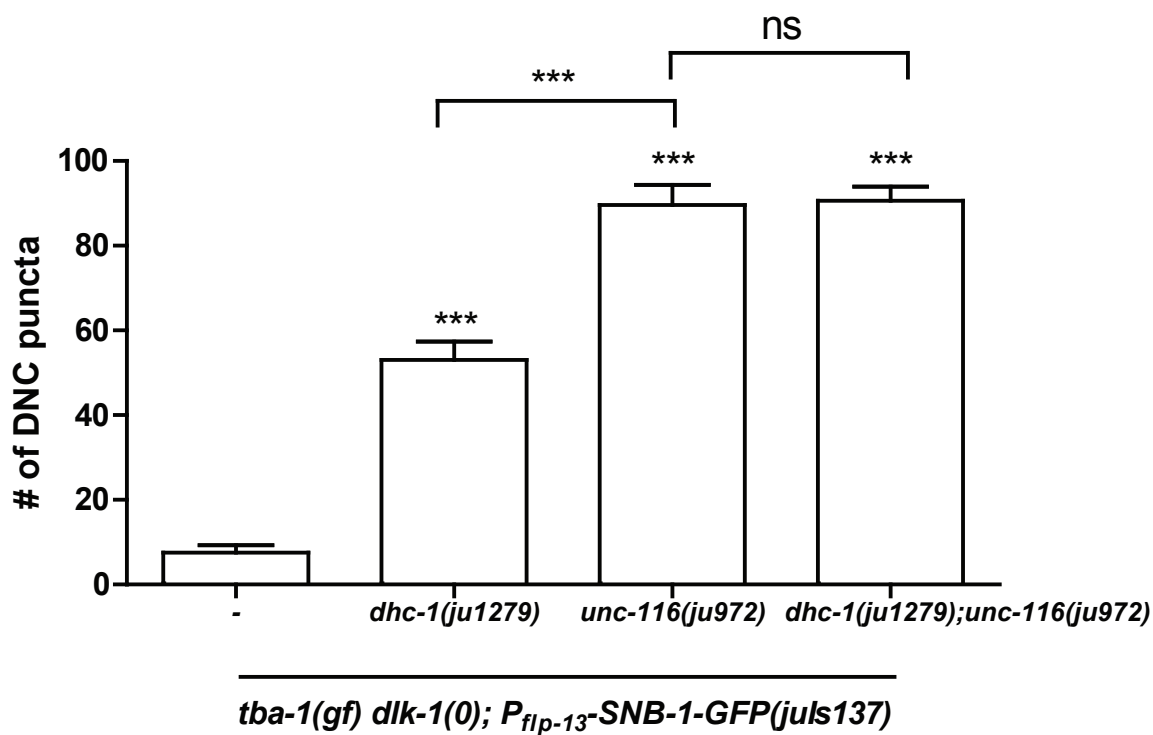

Supplement: S4 Fig — (A, B) Quantification of SNB-1::GFP intensity (Pflp-13-SNB-1-GFP (juIs137)) in the DNC of L4 animals, anaesthetized using 30mM muscimol. Data are mean ± SEM; n = 10 animals per genotype. Statistics: Mann-Whitney test, p-values are displayed on graph. (C) Quantification of synaptic puncta in the DNC of adult animals. Data are mean ± SEM; n>10 animals per genotype. Statistics: One-Way ANOVA followed by Tukey’s posttest; ***p<0.001, ns- not significant. (PDF) [file pgen.1006844.s009.pdf]
