# Supplementary material for: Differential regulation of polarized synaptic vesicle trafficking and synapse stability in neural circuit rewiring in Caenorhabditis elegans
Source: PLoS Genet. 2017 Jun 21;13(6):e1006844. doi: 10.1371/journal.pgen.1006844 (PMC5500376; doi:10.1371/journal.pgen.1006844)
Supplement: S2 Fig — (A) Alignment of sequences of dynein heavy chains from C.elegans, H. sapiens and M. musculus, surrounding the conserved Proline residue that is mutated in ju1279. (B) Bright field images of various dhc-1 alleles (homozygous ju1279 and js319, and heterozygous ju1279/js319) cultured at 25°C. (C, D) Representative kymographs of EBP-2 movement in the adult VNC of the respective genotypes. Red lines indicate EBP-2 comets moving in the anterograde direction. (E, F) Quantification of number of EBP-2 comets (D) and their direction of movement (E) for various genotypes. Data are mean ± SEM; n = number of animals (shown on (E)). Statistics: One-way ANOVA followed by Tukey’s posttest; ***p<0.001, n.s.-not significant. (G) Bright field images of various dnc-4 alleles (homozygous ju933 and or633, and heterozygous ju933/or633) cultured at 25°C. (PDF) [file pgen.1006844.s007.pdf]

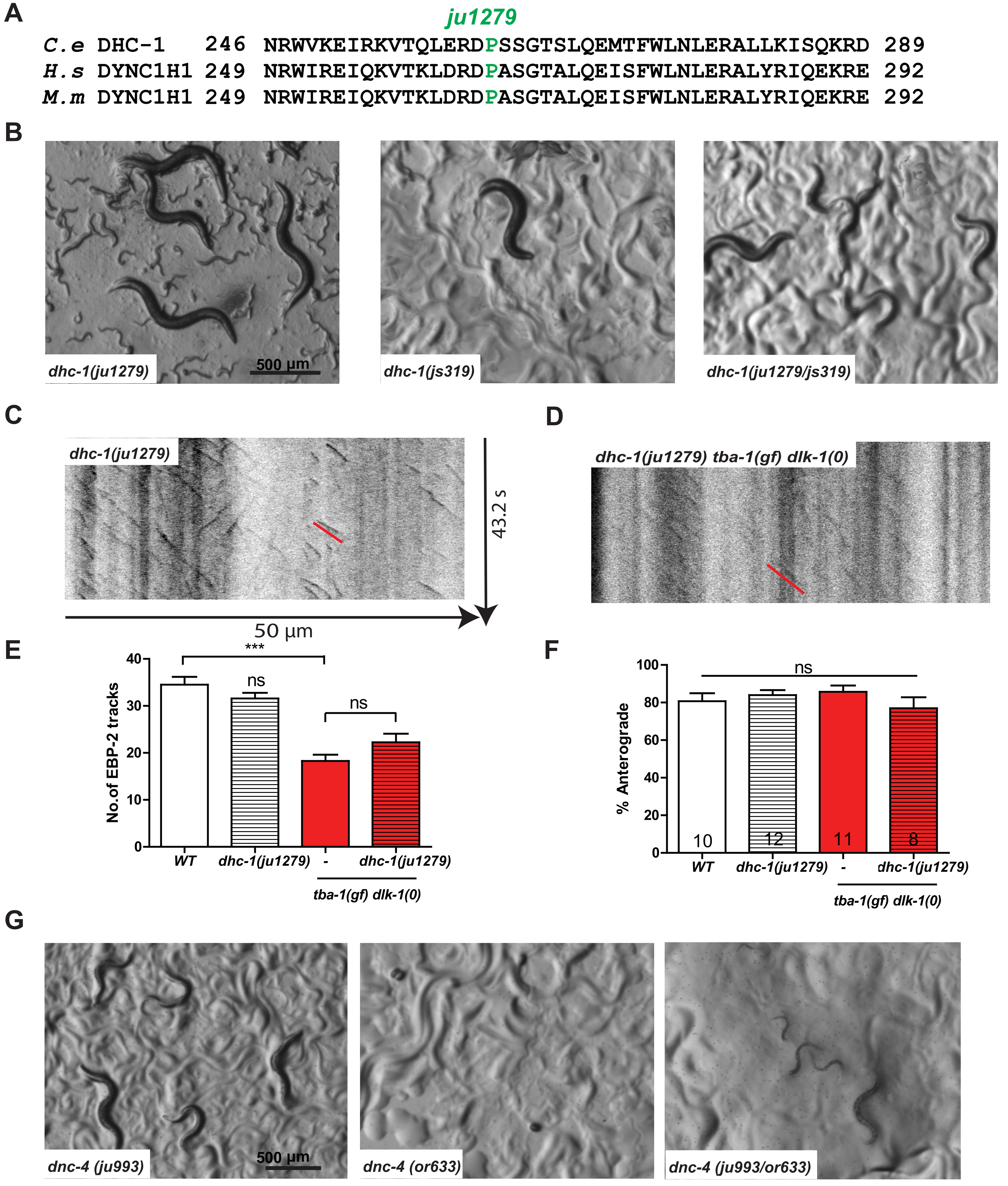
A
ju1279
C.e DHC-1 246 NRWVKEIRKVTQLERDPSSGTSLQEMTFWLNLERALLKISQKRD 289
H.s DYNC1H1 249 NRWIREIQKVTKLDRDPASGTALQEISFWLNLERALYRIQEKRE 292
M.m DYNC1H1 249 NRWIREIQKVTKLDRDPASGTALQEISFWLNLERALYRIQEKRE 292
B
dhc-1(ju1279)
500 µm
dhc-1(js319)
dhc-1(ju1279/js319)
C
dhc-1(ju1279)
43.2 s
50 µm
D
dhc-1(ju1279) tba-1(gf) dlk-1(0)
E
***
ns
ns
No.of EBP-2 tracks
40
30
20
10
0
WT
dhc-1(ju1279)
-
dhc-1(ju1279)
tba-1(gf) dlk-1(0)
F
ns
% Anterograde
100
80
60
40
20
0
10
12
11
8
WT
dhc-1(ju1279)
-
dhc-1(ju1279)
tba-1(gf) dlk-1(0)
G
dnc-4 (ju993)
500 µm
dnc-4 (or633)
dnc-4 (ju993/or633)
